# A New Method to Facilitate Valid and Consistent Grading Cardiac Events in Childhood Cancer Survivors Using Medical Records

**DOI:** 10.1371/journal.pone.0100432

**Published:** 2014-07-09

**Authors:** Elizabeth (Lieke) A. M. Feijen, Helena J. van der Pal, Elvira C. van Dalen, Renee L. Mulder, Edit Bardi, Claudia Kuehni, Wim J. E. Tissing, Leontine C. M. Kremer

**Affiliations:** 1 Department of Pediatric Oncology, Emma Children’s Hospital/Academic Medical Center Amsterdam, Amsterdam, the Netherlands; 2 Department of Oncology, Academic Medical Center Amsterdam, Amsterdam, the Netherlands; 3 2^nd^ Department of Pediatrics, Semmelweis University, Budapest, Hungary; 4 Department of Pediatric Oncology, Institute of Social and Preventive Medicine, University of Bern, Bern, Switzerland; 5 Department of Pediatric Oncology, University of Groningen, University Medical Center Groningen, Groningen, The Netherlands; University of York, United Kingdom

## Abstract

**Background:**

Cardiac events (CEs) are among the most serious late effects following childhood cancer treatment. To establish accurate risk estimates for the occurrence of CEs it is essential that they are graded in a valid and consistent manner, especially for international studies. We therefore developed a data-extraction form and a set of flowcharts to grade CEs and tested the validity and consistency of this approach in a series of patients.

**Methods:**

The Common Terminology Criteria for Adverse Events version 3.0 and 4.0 were used to define the CEs. Forty patients were randomly selected from a cohort of 72 subjects with known CEs that had been graded by a physician for an earlier study. To establish whether the new method was valid for appropriate grading, a non-physician graded the CEs by using the new method. To evaluate consistency of the grading, the same charts were graded again by two other non-physicians, one with receiving brief introduction and one with receiving extensive training on the new method. We calculated weighted Kappa statistics to quantify inter-observer agreement.

**Results:**

The inter-observer agreement was 0.92 (95% CI 0.80–1.00) for validity, and 0.88 (0.79–0.98) and 0.99 (0.96–1.00) for consistency with the outcome assessors who had the brief introduction and the extensive training, respectively.

**Conclusions:**

The newly developed standardized method to grade CEs using data from medical records has shown excellent validity and consistency. The study showed that the method can be correctly applied by researchers without a medical background, provided that they receive adequate training.

## Introduction

Due to the improvement in treatment protocols and new treatment modalities survival from childhood cancer is currently around 80% [1]. Inherent to this improvement in childhood cancer survival is the growing population of childhood cancer survivors (CCS). However, around 75% of survivors will have at least one late adverse effect (e.g. endocrine, neurologic or psychosocial late adverse effects) induced by the cancer treatment [2]. Knowledge of the incidence and risk factors for specific late adverse effects is essential, as it contributes to optimal follow-up care for survivors and recommendations for less toxic treatments for future childhood cancer patients. Frequent late effects within CCS are cardiac events (CE), such as heart failure, ischemia, pericarditis, valvular disease and arrhythmia, all of which cause long-term morbidity and early mortality [3], [4]. After a median follow-up time of more than thirteen years, the cumulative incidence of symptomatic heart failure is 1.7–2%, ischemia 0.44–0.7%, pericarditis 0.14–1.3%, valvular disease 0.44–1.6% and arrhythmia 0.66% [3], [5].

A major limitation in current studies of CEs is the lack of uniform outcome definitions for the events in question. Definitions vary between research groups; even those within the same country. In addition, the CEs are often graded by several physicians (from different specialities), based on expert opinion, and without a clear grading protocol [3], [5]–[8]. For example, in a previous study of van der Pal et al. [3] two authors (both physicians) graded CEs using the Criteria for Adverse Events (CTCAE) version 3.0 (heart failure, ischemia, pericarditis, valvular disease and arrhythmia grade 3–5) consulting a cardiologist when uncertain [3]. On the other hand, Mulrooney et al. [5] used self-reported CEs. Survivors were asked if they had ever been told by a doctor or other healthcare professional, that they have, or have had, a CE (i.e. heart failure, myocardial infarction, valvular abnormalities or pericardial disease). Within this study the severity of the CEs could not be established. Therefore, the lack of uniform outcome definitions for CEs makes it impossible to compare the results of existing studies and to summarize the evidence, thus making it difficult to make recommendations for clinical practice. Furthermore, Atkinson et al. [9] showed that agreement between different clinicians when reporting adverse events is “moderate” at best, even when clear outcome definitions (i.e. the CTCAE) are used. This study shows that even uniform outcome definitions for CEs are not sufficient and that there is a need for a clear grading protocol.

At this moment a large pan-European study is being conducted; PanCareSurFup (PanCare Childhood and Adolescent Cancer Survivor care and Follow-up studies (PCSF)). One of the main objectives of PCSF is to identify CCS who have developed a symptomatic CE. Seven different European countries (the United Kingdom, France, Italy, Switzerland, Slovenia, Hungary and the Netherlands) will contribute cardiac data to this study and the incidence and absolute risk of cardiac disease among 5-year CCS will be determined. Furthermore, a nested case-control study will be undertaken to investigate the nature of the dose-response relationship between cumulative dose of specific anti-cancer drugs, cumulative dose of irradiation, and the risk of a CE. Outcome assessors will have different specialties, i.e. physicians and non-physicians (e.g. data managers or research nurses). To adequately analyse the data from the different countries the CEs need to be graded and validated in a uniform manner across Europe.

The aim of this study was to test the validity and consistency of a newly developed data-extraction form in combination with a flowchart to grade CEs in a group of CCS with a known CE.

## Methods

### Study population

We included CCS with a previously defined symptomatic CE from the cohort described in van der Pal et al. 2012 [3]. This cohort consisted of 1362 5-year CCS who were diagnosed with childhood cancer in the Emma’s Children Hospital/Academic Medical Center between January 1966 and January 1996. Seventy-two survivors were suspected of a symptomatic CE during follow-up. After careful review forty-two patients were coded as a symptomatic CE (CTCAEv3.0 grade ≥3) and 30 patients were coded as an asymptomatic CE (CTCAEv3.0 grade ≤2). Our outpatient clinic for follow-up after treatment for childhood cancer was reviewed by the Institutional Review Board of the Academical Medical Center in Amsterdam and the study was deemed as patient care and was therefore exempt from the need for ethical approval. Throughout patient care, acquired outcomes are used for scientific research to evaluate care. Additionally, CCS gave informed consent for data collection from the medical records. Patient records were anonymized and de-identified prior to analysis.

### The new method: data-extraction form/flowchart method for CEs

**Figure 1:**
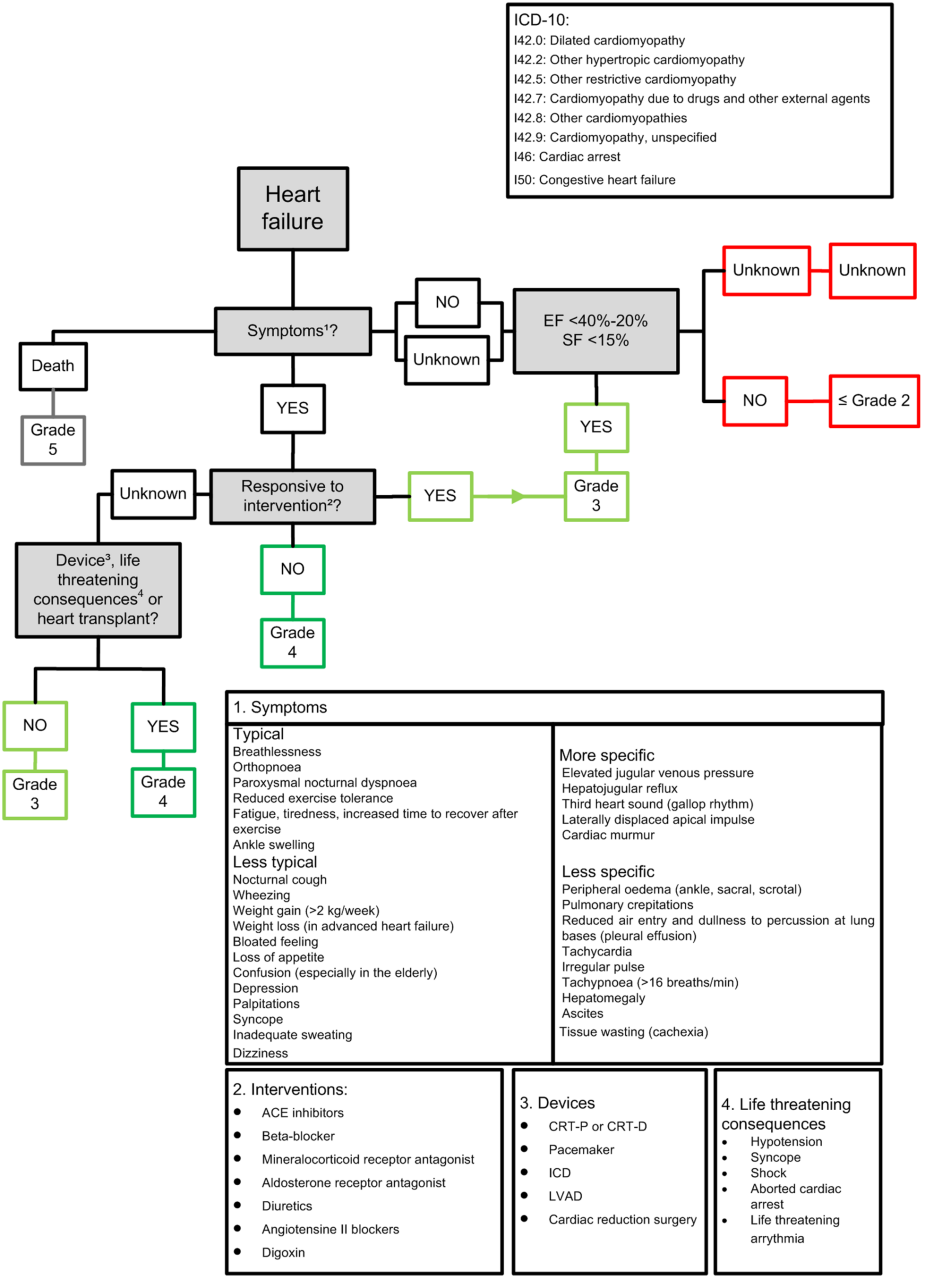
Flowchart heart failure. 1a. The first question is *“Symptoms^1^?”*, “number 1″ refers to block 1 under the step diagram, in this block symptoms of heart failure are shown, so the user knows which symptoms could occur; When the answer is “NO”. 1b. Is the *“EF<40%-20% or the FS<15%”* 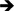 YES grade 3 heart failure. 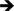 NO grade ≤2 heart failure. When the answer is “YES” 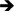 Go to question “Responsive to intervention^2^?” 2. Question *“Responsive to intervention^2^?”*, block 2 in which common interventions for heart failure are shown; When the answer is “NO” 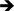 grade 4 heart failure. When the answer is “YES”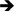 grade 3 heart failure. When it is “UNKNOWN” 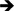 go to question “ *Device^3^, life threatening consequences^4^ or heart transplant?”*. 3. Question “ Device^3^, life threatening consequences^4^ or heart transplant?”, block 3 under the step diagram, in this block devices used as treatment for heart failure are shown. In block 4 the life threatening consequences associated with heart failure are stated; When the answer is “NO” 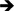 grade 3 heart failure. When the answer is “YES”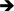 grade 4 heart failure. Ref. [10], [11]. ICD-10 = International classification of disease version 10. EF = ejection fraction. SF = shortening fraction. CRT-P or D = cardiac resynchronisation therapy pacemaker or defibrillator. ICD = implantable cardioverse defibrillator. LVAD = left ventricular assistance device.

We developed a standardized data-extraction form (see SI 1), a set of flowcharts (one for each CE, i.e. heart failure, ischemia, pericarditis, valvular disease and arrhythmia; see SI 1), a manual with background information and a training presentation. The method is developed to distinguish between a CE of grade ≤2 and grade 3, 4 and 5. Grade ≤2 is predominantly asymptomatic. The method consists of two steps; 1) extraction of all relevant information from the available medical records, questionnaire (patient or physician) or interview using the standardized data extraction form and 2) assignment of a grade to the CEs using the appropriate flowchart. In Figure 1 the flowchart of heart failure is shown as an example. Each flowchart is constructed in the same manner; a step diagram and clarifying text blocks. We used a combination of the CTCAEv3.0 and CTCAEv4.0 for the definitions of CEs (see Table 1). Besides the data-extraction form and flowcharts we wrote a manual, including background information on the different CEs, and an extensive explanation on the use of the method (see File S1, Table S1, Figure S1–S5). Finally outcome assessors attended a presentation (see Presentation S1) to explain the method in more detail with the use of examples.

**Table 1:** Definitions of cardiac events (using CTCAEv3.0 and CTCAEv4.0).

	Grade 3	Grade 4	Grade 5
Heart failure	Symptomatic CHF responsiveto intervention, orEF<40%-20%, or SF<15%	Refractory CHF or poorly controlled;EF<20%; intervention such as ventricularassist device, ventricular reductionsurgery, or heart transplant indicated;life threatening consequences*	Death due toheart failure
Ischemia	Symptomatic and testing consistentwith ischemia or unstable angina orintervention needed	Myocardial infarction; lifethreatening consequences*	Death due toischemia
Pericarditis	With physiological consequences(e.g. pericardial constriction orpericardial effusion)	With life threateningconsequences (e.g. hemodynamiccomprise)	Death due topericarditis
Valvularmdisease	Symptoms of severe regurgitationor stenosis, symptoms controlledwith interventions	Life threatening consequences orintervention (e.g. valvereplacement or valvuloplasty)indicated	Death due tovalvular disease
Arrhythmia	Symptomatic and incompletelymedically controlled orcontrolled with device (e.g.pacemaker, ICD or CRT)	Life threatening consequences(e.g. arrhythmia associated withCHF, hypotension, syncope, shock)	Death due to anarrhythmia

*as reported in the Criteria for Adverse Events (CTCAE)v4.0.

CHF = congestive heart failure.

EF = ejection fraction.

SF = shortening fraction.

ICD = implantable cardioverse defibrillator.

CRT = cardiac resynchronisation therapy.

Note: If a CE doesn’t comply with any of these criteria, it should be graded as grade ≤2.

### Validity and consistency of the data-extraction form/flowchart method for CEs

**Figure 2:**
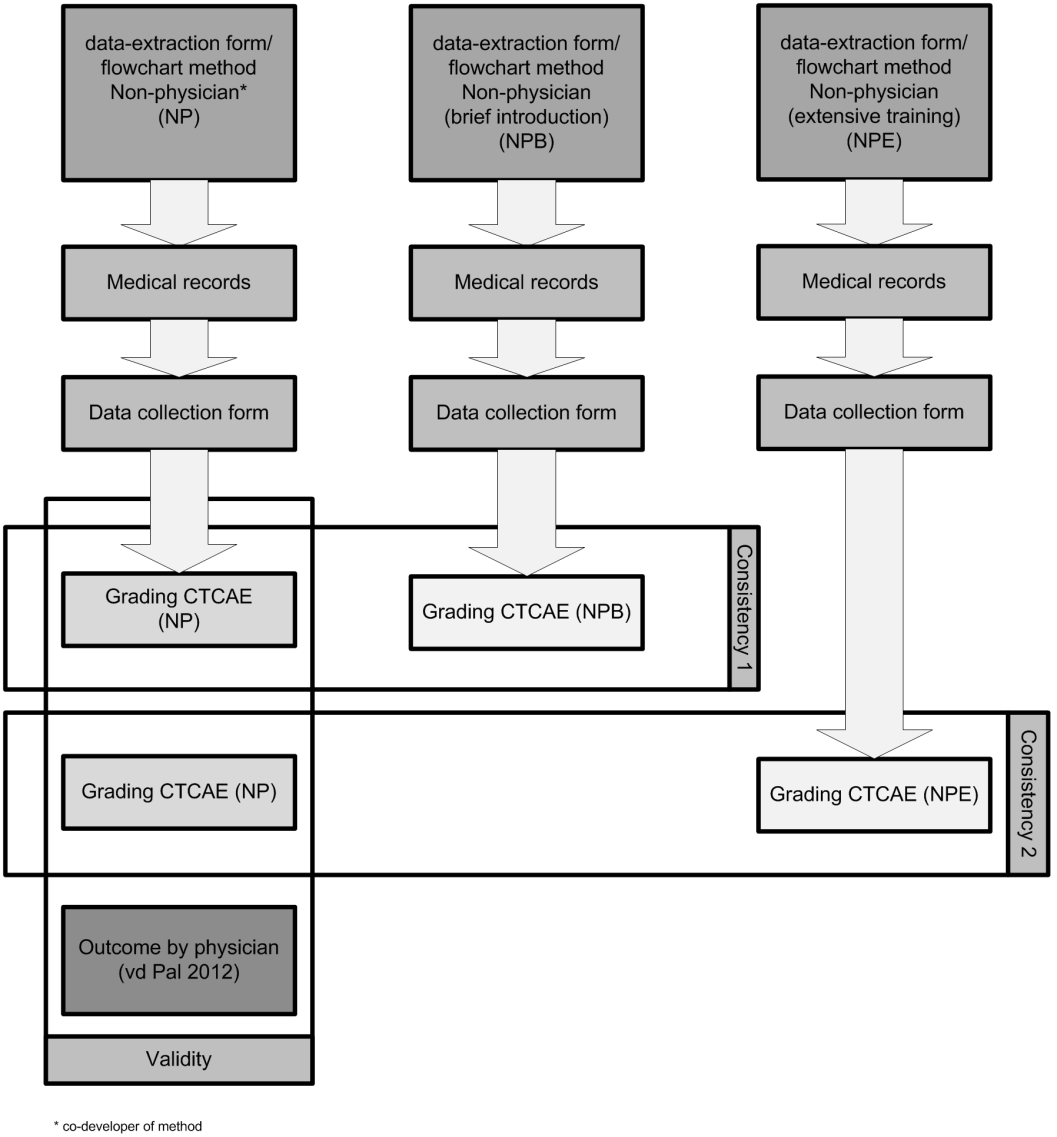
Methodology of testing the validity and consistency of the data-extraction form/flowchart method for CEs.

In Figure 2 the methodology for testing the validity and consistency is schematically shown.

The validity of our new method was tested by comparing the CE grading outcome of the physician of the forty randomly selected patients from the seventy-two CCS from a previous study [3], with the new grading outcome using the data-extraction form/flowchart method as graded by a non-physician. This non-physician had been involved in the development of the new method, but could be considered as a non-physician who had received an extensive training.

The consistency of the new method was tested by comparing the grading of the non-physician involved in the development of the new method with the grading of two other non-physicians, of whom one who had received a brief introduction to the method, based on the text below the flowcharts, and a second had received extensive training on the new method by means of the full manual and a presentation with an example case-study. In the first consistency test we compared the grading of the non-physician involved in the development of the method with the grading of the non-physician who received solely a brief introduction. In the second consistency test we compared the grading of the non-physician involved in the development of the method with the grading of the non-physician who received an extensive training. In this way we were able to test the robustness of the new method as well as the additional value of the extensive training. The first consistency test shows if the method on its own is sufficient for consistent grading of cardiac events. By comparing the results of the first consistency test with those of the second consistency test we can determine the additional value of the extensive training. The non-physicians were blinded for the results of the physician and the other non-physicians.

### Data extraction

The necessary information was taken from medical charts. The medical charts were readily available since they were already collected for the study of van de Pal et al. [3]. To properly grade the CE information was needed on symptoms, diagnostic tests, medication and surgery. The goal was to get complete data on all those subjects for each CE.

### Statistical analysis

To determine the agreement between the different outcome assessors we calculated a weighted Kappa [12], [13]. The weighted Kappa is used when there are several ordered grades and is calculated with the following formula: (probability of observed matches - probability of expected matches)/(1 - probability of expected matches). The disagreements are weighted according to their squared distance from perfect agreement. R was used to calculate the weighted Kappa and 95% confidence intervals [14]. Values of Kappa between 0.40 and 0.59 are considered to reflect a fair agreement, between 0.60 and 0.74 to reflect a good agreement and 0.75 or more to reflect an excellent agreement [15].

## Results

### Study population

The median age of the forty persons (18 females), was 9.9 years (range 0.6–17.2) at the time of cancer diagnosis and 31.1 years (range 13.5–46.4) at the time of follow-up, with a median follow-up time of 21.6 years (range 5.1–36.1) since diagnosis. Fifteen subjects (37.5%) had received anthracyclines alone, ten subjects (25%) radiotherapy involving the heart region and eleven subjects (27.5%) anthracyclines and radiotherapy involving the heart region. Four subjects (10%) had not received any known cardiotoxic treatment. Nineteen subjects (47.5%) had been diagnosed with heart failure (grade ≤2 n = 6, grade 3 n = 8, grade 4 n = 2, grade 5 n = 3) as first occurring CE, three subjects (7.5%) with ischemia (grade 3 n = 2, grade 4 n = 1), one subject (2.5%) with pericarditis (grade 4 n = 1), fourteen subjects (35%) with valvular disease (grade ≤2 n = 11, grade 3 n = 2, grade 4 n = 1) and three subject (7.5%) with arrhythmia (grade ≤2 n = 1, grade 3 n = 2).

### Validity and consistency of the data-extraction form/flowchart method for CEs

The results of the validity test are shown in Table 2. The inter-observer agreement for the comparison between the grading of the main non-physician and the grading of the physician in the previous study [3] was 0.92 (0.80–1.00). Three CEs were graded differently: two of them were graded as grade 3 by the non-physician and as grade ≤2 in the previous study [3]. The third CE was graded differently due to incomplete medical records.

**Table 2:** Result for validity (physician (P) vs non-physician (NP).

		P		
		Grade ≤2	Grade 3	Grade 4
**NP**	**Grade ≤2**	***17***	0	0
	**Grade 3**	2*	***12***	0
	**Grade 4**	0	0	***5***

*Grade unknown n = 1 not included.

**Kappa = 0.92 (0.80–1.00).**

The results of the two consistency assessments are presented in Table 3 and 4. The inter-observer agreement for the comparison between the non-physician involved in the development of the new method and the results of a non-physician who only received a brief introduction of the method was 0.88 (0.79–0.98). Eight CEs were graded differently (Table 2b), but always in an adjacent severity category.

**Table 3:** Result first consistency assessment (non-physician (NP) vs non-physician (brief introduction) (NPB)).

		NP		
		Grade ≤2	Grade 3	Grade 4
**NPB**	**Grade ≤2**	***14***	2	0
	**Grade 3**	3	***12***	3
	**Grade 4**	0	0	***2***

**Kappa = 0.88 (0.79–0.98).**

**Table 4:** Result second consistency assessment (non-physician (NP) vs non-physician (extensive training) (NPE)).

		NP		
		Grade ≤2	Grade 3	Grade 4
**NPE**	**Grade ≤2**	***17***	1	0
	**Grade 3**	0	***11***	0
	**Grade 4**	0	0	***5***

**Kappa = 0.99 (0.96–1.00).**

The inter-observer agreement for the comparison between the non-physician involved in the development of the new method and the results of a non-physician who received extensive training on the new method by means of the above mentioned manual and presentation was 0.99 (0.96–1.00). Only one CE was graded differently (Table 2c).

## Discussion

This study demonstrates that our new standardised method for grading CEs in CCS using a data-extraction form and a set of flowcharts is valid and consistent. The random selection of the known cases resulted in a variation of CEs including different diagnoses and different levels of severity; therefore all five flowcharts were tested in this current study. With this method non-physicians can score CEs in an accurate manner. However, the best results from non-physicians were achieved when extensive training was given. Not all relevant data was extracted by the non-physician who received only a brief introduction of the method, resulting in a lower inter-observer agreement. The non-physician who received only a brief introduction of the method also had a limited knowledge of CEs. None of the previous studies focussing on CEs after cancer treatment had used a standardised method for the definition of the outcomes as described in this paper. The extraction form describes very specifically the information that is essential in order to grade the CE. This information can often be extracted from test results or letters, which are easy to interpret. Therefore, a strength of our method is that the invested time for retrieving necessary information for grading the CEs is minimal.

A limitation of this current study is that although the data-extraction/flowchart method may be used with several types of data (e.g. questionnaire, interviews or information from doctors), the current study only validated the method through the use of medical charts. With this study we wanted to confirm in principle that the data-extraction form/flowchart method is a valid and consistent method of grading a CE. The completeness in medical charts, compared to other sources of data, can be considered a benefit for this purpose. The external validity of the results of this study has not yet been tested in other institutes.

PanCareSurFup is a large pan-European study, of which one of the main objectives is to collect symptomatic CEs. Based on our findings, we believe that the data-extraction form/flowchart method can be safely used to consistently grade the CEs, across the different European countries. The current method is developed for CEs, but the CTCAE is available for adverse events of different organ systems. A similar method could be developed for different other organs systems, which could then be applied in collaborative research.

We conclude that our newly developed method is a valid and consistent way to grade CEs. This method can be used by assessors with different medical background, provided that they receive proper instruction about the method, for which the manual and the training presentation are available.

## Supporting Information

Figure S1 Flowchart Heart failure.
(TIF)

Figure S2 Flowchart Ischemia.
(TIF)

Figure S3 Flowchart Pericarditis.
(TIF)

Figure S4 Flowchart Valvular disease.
(TIF)

Figure S5 Flowchart Arrhythmia.
(TIF)

Table S1 Extraction form. All this data should be used for grading the cardiac event, with the help of the flowchart.
(DOC)

File S1 Manual text including background information on the different CEs, and an extensive explanation on the use of the method.
(DOC)

Presentation S1 Training presentation.
(PPS)

## References

[1] Gatta G, Zigon G, Capocaccia R, Coebergh JW, Desandes E, et al (2009) Survival of European children and young adults with cancer diagnosed 1995–2002. Eur J Cancer 45: 992–1005. 19231160 10.1016/j.ejca.2008.11.042

[2] Geenen MM, Cardous-Ubbink MC, Kremer LCM, Van den Bos C, Van der Pal HJH, et al (2007) Medical assessment of adverse health outcomes in long-term survivors of childhood cancer. J Am Med Assoc 297: 2705–15. 10.1001/jama.297.24.2705 17595271

[3] Van der Pal HJ, Van Dalen EC, Van Delden E, Van Dijk IW, Kok WE, et al (2012) High risk of symptomatic cardiac events in childhood cancer survivors. J Clin Oncol 30: 1429–37. 22473161 10.1200/JCO.2010.33.4730

[4] Reulen RC, Winter DL, Frobisher C, Lancashire ER, Stiller CA, et al (2010) Long-term Cause-Specific Mortality Among Survivors of Childhood Cancer. J Am Med Assoc 304: 172–9. 10.1001/jama.2010.923 20628130

[5] Mulrooney DA, Yeazel MW, Kwashima T, Mertens AC, Mitby P, et al (2009) Cardiac outcomes in a cohort of adult survivors of childhood and adolescent cancer: retrospective analysis of the Childhood Cancer Survivor Study cohort. Br Med J 339: b4606. 19996459 10.1136/bmj.b4606 PMC3266843

[6] Armenian SH, Sun C, Francisco L, Steinberger J, Kurian S, et al (2008) Late Congestive Heart Failure After Hematopoietic Cell Transplantation. J Clin Oncol 28: 5537–43. 10.1200/JCO.2008.17.7428 PMC2651101 18809605

[7] Green DM, Grigoriev YA, Takashima JR, Norkool PA, D’Angio GJ, et al (2001) Congestive Heart Failure After Treatment for Wilms’ Tumor: A Report From the National Wilms’ Tumor Study Group. J Clin Oncol 19: 1926–34. 11283124 10.1200/JCO.2001.19.7.1926

[8] Van Dalen EC, Van der Pal HJH, Kok WEM, Caron HN, Kremer LCM (2006) Clinical heart failure in a cohort of children treated with anthracyclines: A long-term follow-up study. Eur J Cancer 42: 3191–98. 16987655 10.1016/j.ejca.2006.08.005

[9] Atkinson TM, Li Y, Coffey CW, Sit L, Shaw M, et al (2012) Reliability of adverse symptom event reporting by clinicians. Qual Life Res 21: 1159–64. 21984468 10.1007/s11136-011-0031-4 PMC3633532

[10] Dickstein K, Cohen-Solal A, Filippatos G, McMurray JJV, Ponikowski P, et al (2008) ESC Guidelines for the diagnosis and treatment of acute and chronic heart failure 2008. The Task Force for the Diagnosis and Treatment of Acute and Chronic Heart Failure 2008 of the European Society of Cardiology. Developed in collaboration with the Heart Failure Association (HFA) of the ESC and endorsed by the ESICM. Eur Heart J 29: 2388–442. 18799522 10.1093/eurheartj/ehn309

[11] McMurray JJV, Adamopoulos S, Anker SD, Auricchio A, Bohm M, et al. (2012) ESC Guidelines for the diagnosis and treatment of acute and chronic heart failure 2012. The Task Force for the Diagnosis and Treatment of Acute and Chronic Heart Failure 2012 of the European Society of Cardiology Developed in collaboration with the Heart Failure Association (HFA) of the ESC. Eur Heart J doi:10.1093/eurheartj/ehs104. 10.1093/eurjhf/hfs105 22828712

[12] Fleiss JL, Cohen J, Everitt BS (1969) Large sample standard errors of kappa and weighted kappa. Psychol Bull 72: 323–27.

[13] Cohen J (1968) Weighted kappa: nominal scale agreement with provision for scaled disagreement or partial credit. Psychol Bull 70: 213–20. 19673146 10.1037/h0026256

[14] R Development Core Team (2008) R: A language and environment for statistical computing. R Foundation for Statistical Computing, Vienna, Austria. ISBN 3-900051-07-0, URL http://www.R-project.org.

[15] Orwin RG (1994) Evaluating coding decisions. In: Cooper H, Hedges LV, editors. The Handbook of Research Synthesis. New York (NY): Russell Sage Foundation. 177–200.

