# Supplementary figures and images for: A New Method to Facilitate Valid and Consistent Grading Cardiac Events in Childhood Cancer Survivors Using Medical Records
Source: PLoS One. 2014 Jul 9;9(7):e100432. doi: 10.1371/journal.pone.0100432 (PMC4090125; doi:10.1371/journal.pone.0100432)

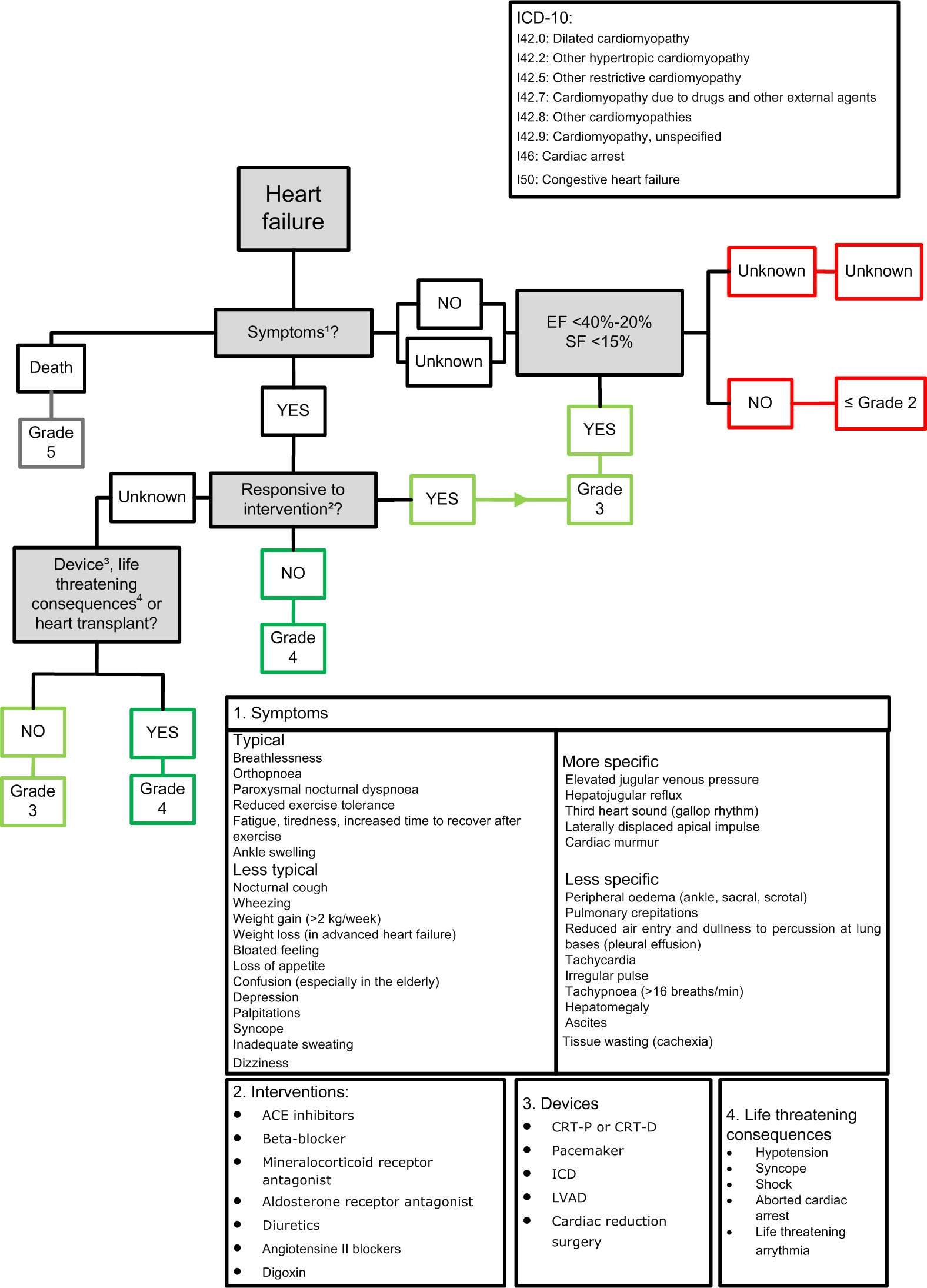

Supplement: Figure S1 — Flowchart Heart failure. (TIF) [file pone.0100432.s001.tif]

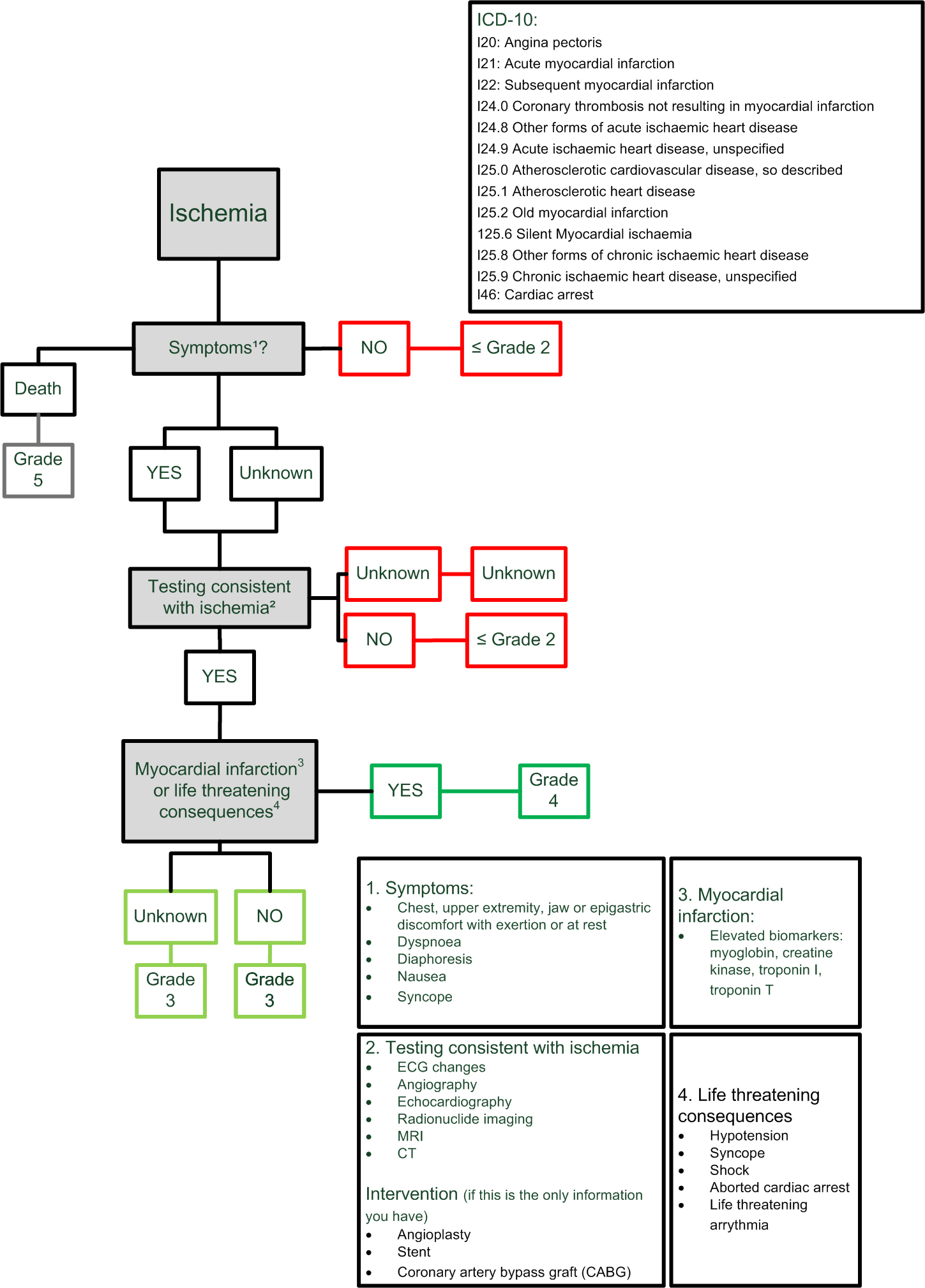

Supplement: Figure S2 — Flowchart Ischemia. (TIF) [file pone.0100432.s002.tif]

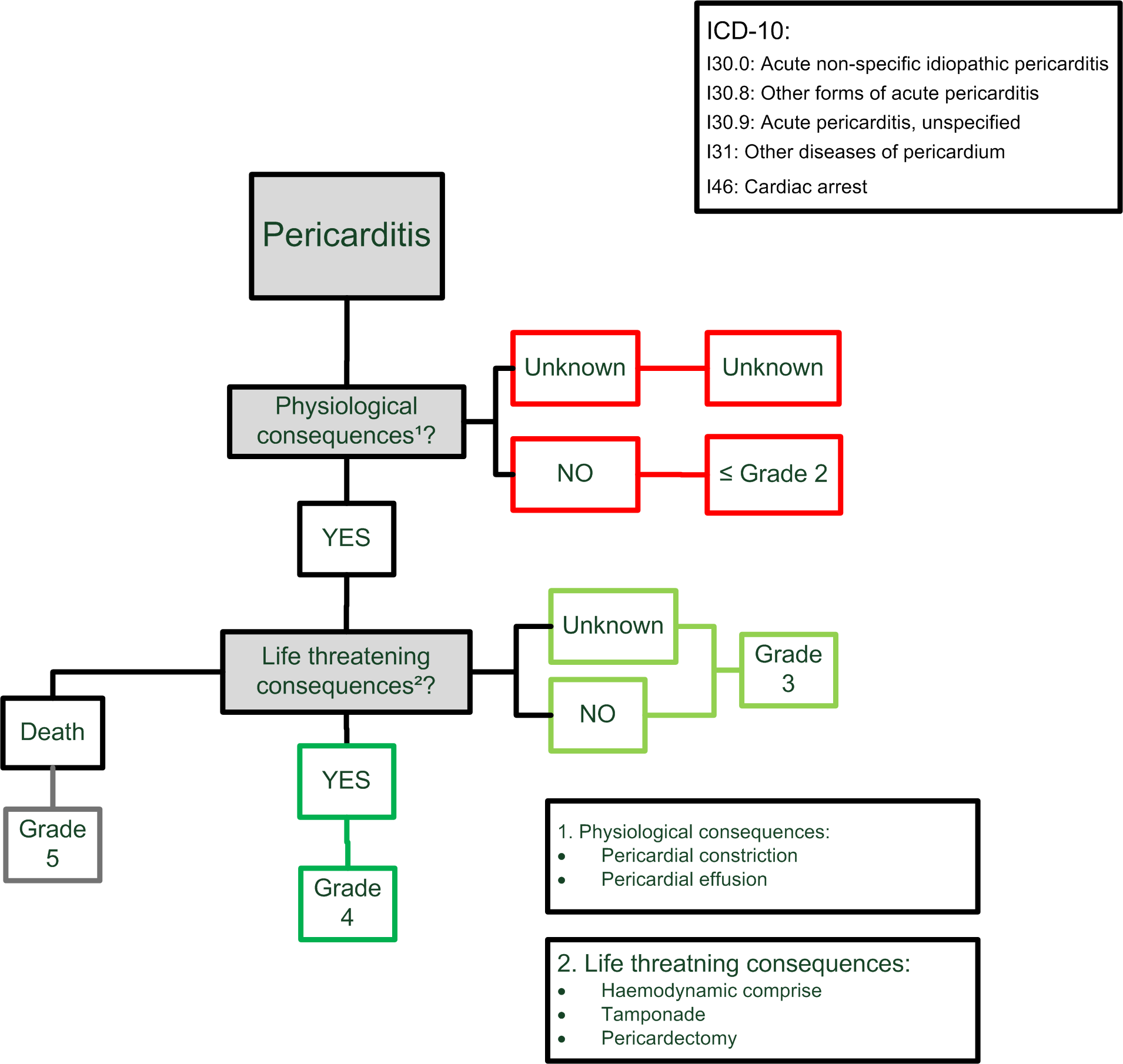

Supplement: Figure S3 — Flowchart Pericarditis. (TIF) [file pone.0100432.s003.tif]

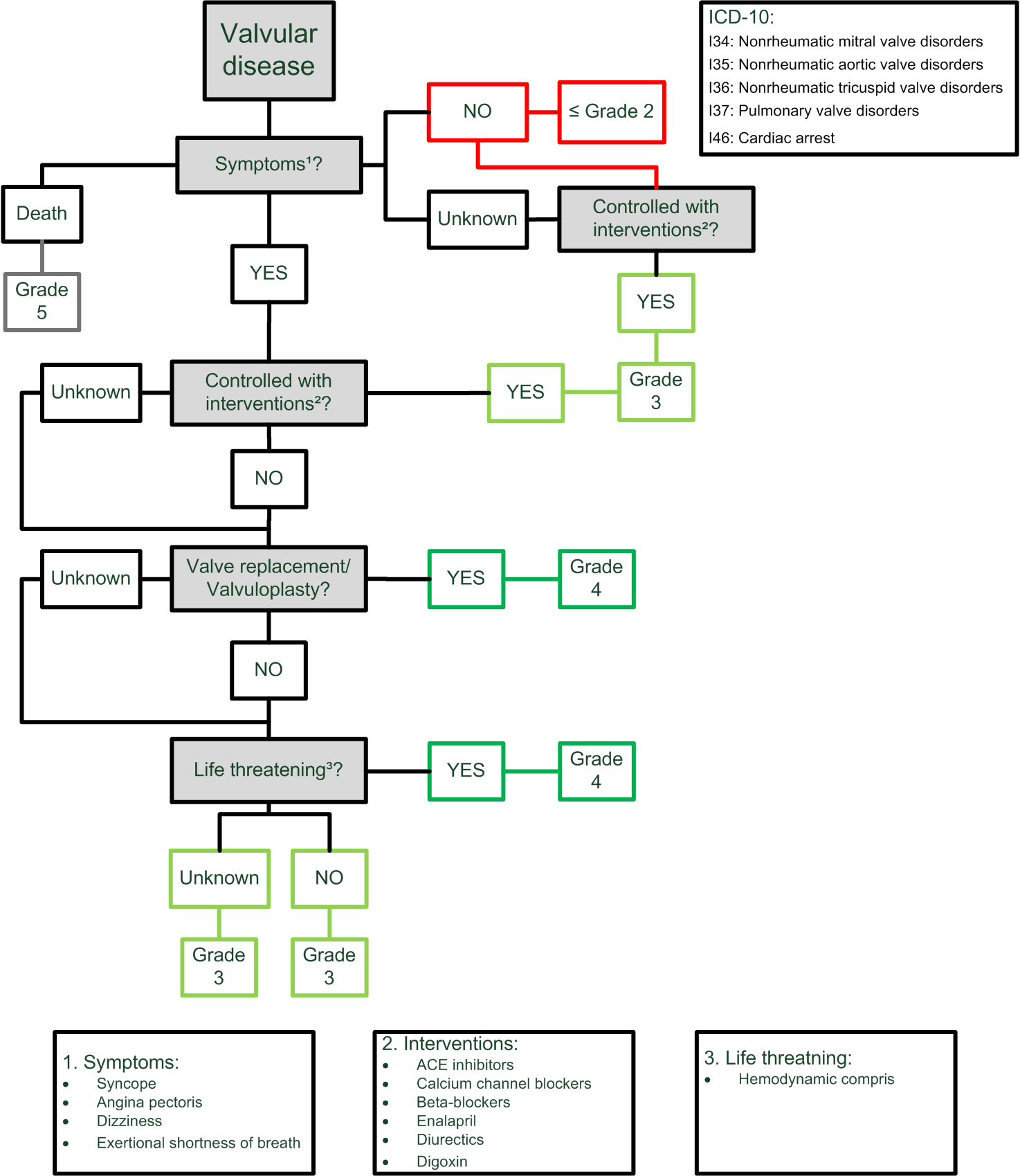

Supplement: Figure S4 — Flowchart Valvular disease. (TIF) [file pone.0100432.s004.tif]

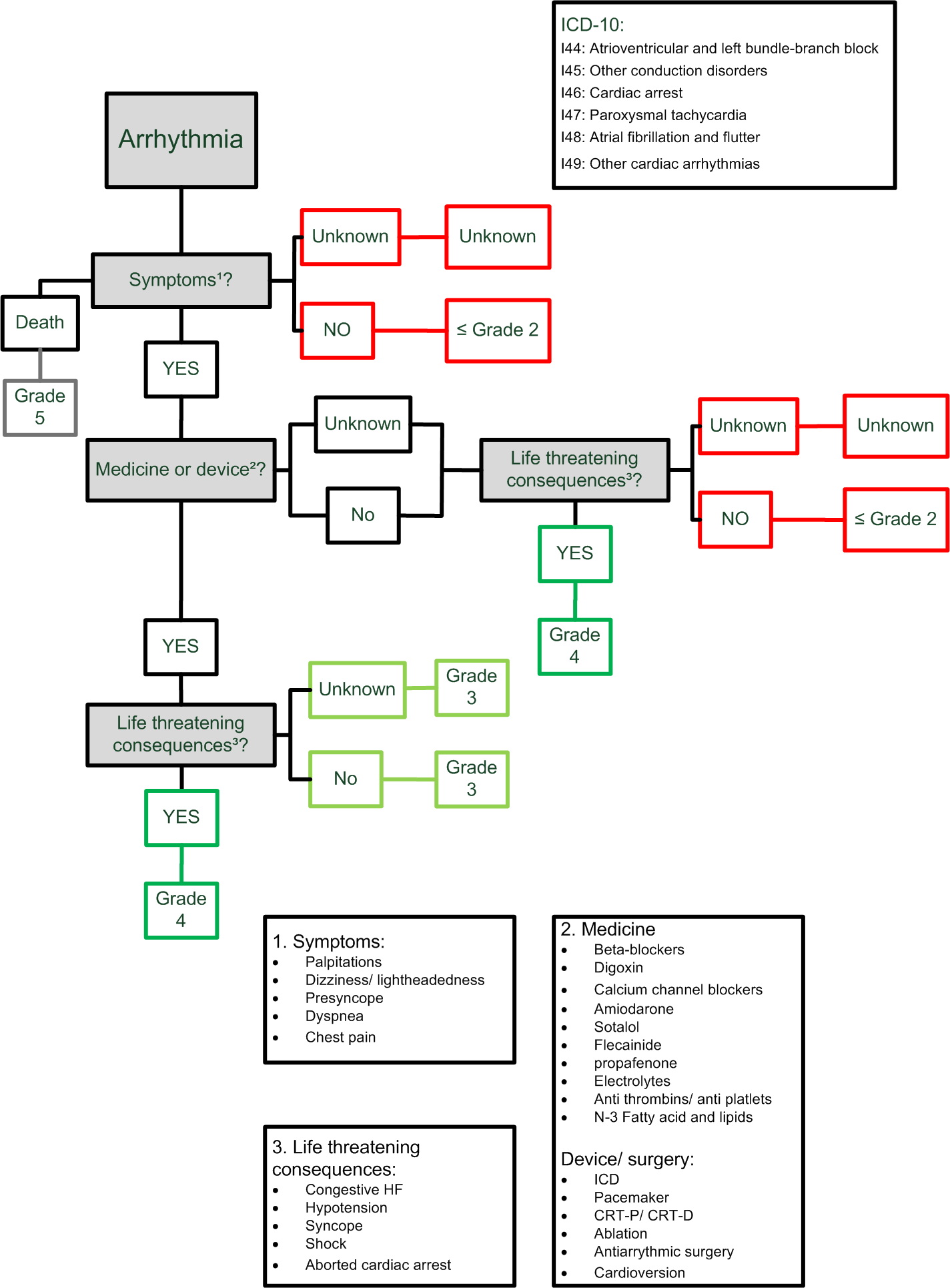

Supplement: Figure S5 — Flowchart Arrhythmia. (TIF) [file pone.0100432.s005.tif]
