# Supplementary material for: A New Method to Facilitate Valid and Consistent Grading Cardiac Events in Childhood Cancer Survivors Using Medical Records
Source: PLoS One. 2014 Jul 9;9(7):e100432. doi: 10.1371/journal.pone.0100432 (PMC4090125; doi:10.1371/journal.pone.0100432)
Supplement: Table S1 — Extraction form. All this data should be used for grading the cardiac event, with the help of the flowchart. (DOC) [file pone.0100432.s006.doc]

| Name |  |
| --- | --- |
| Date |  |
| ID nr |  |
| Month of birth |  |
| Year of birth |  |
| Month of incidence |  |
| Year of incidence |  |
| Month of follow-up |  |
| Year of follow-up |  |

| Method of ascertainment |
| --- |
| - Questionnaire health care professional - Questionnaire to survivor or their family, friends or social carer - (Outpatient) Clinic visit - Medical records - Other_______________ |

| Cardiac event | Description of cardiac event |
| --- | --- |
| Cardiac event nr.:    - Heart failure date_______ - Ischemia date_______ - Pericarditis date_______ - Valvular disease date_______ - Arrhythmia date_______ |  |
|  | Other conditions (yes/ no) |
|  |  |

| Symptoms (yes/no) | Additional diagnostic results (yes/ no) |
| --- | --- |
|  |  |
| Life threatening symptoms (yes/ no) |
|  |

| Medication (yes/ no)  Responsive to medication (yes/ no) | | | Cardiac surgery + date (yes/ no) | |
| --- | --- | --- | --- | --- |
| Name | Dose | Since - until | Type | Date |
|  |  |  |  |  |

| Outcome flowchart:______________________________  Method of validation: Medical records/ Treating physician/ other__________ |
| --- |

Explanation:

- Name: Name of the person who is validating.
- Date: Date of validation
- ID nr: PanCareSurFup number which is given to the patient in WP1.
- Month of birth: The month in which the patient is born.
- Year of birth: The year in which the patient is born.
- Month of incidence: The month in which the first primary tumour is diagnosed.
- Year of incidence: The year in which the first primary tumour is diagnosed.
- Month of follow-up: The month until there is follow-up for the patient, concerning cardiac disease (last contact moment).
- Year of follow-up: The year until there is follow-up for the patient, concerning cardiac disease (last contact moment).
- Method of ascertainment: How was the (extra) information collected?
- Cardiac event: which cardiac event, and on what date. For every cardiac event there should be a separate form.
- Cardiac event nr.: Fill in the number of the cardiac event, first event is the earliest event.
- Description of the cardiac event: The cardiac event should be elaborated on, e.g. if it is valvular disease, which valve and whether it is reguration or stenosis. It should also be stated if the patient is responsive to the given treatment.
- Other condition: Fill in if the patient ever had/ has any other conditions (yes/ no). If the patient ever had/ has other severe conditions this should be described in the square, also congenital heart defects can be stated here.
- Symptoms: Fill in if the patient ever had/ has any symptoms (yes/ no). In the square it should state which symptoms.
- Life threatening symptoms: Fill in if the patient ever had/ has any life threatening symptoms (yes/ no). In the square it should state the life threatening symptoms like hemodynamic comprise, life threatening arrhythmias, survived cardiac arrest etc.
- Additional diagnostic results: Fill in if the patient ever had any diagnostic tests (yes/ no). Every echo/ ecg/ blood or other test which is conducted in order to diagnose the cardiac disease.
- Medication: Fill in if the patient ever used/ uses any medication (yes/ no). Which medication (generic name) is used for the cardiac disease, what is the dose and since when( – until when) does the patient take this medication.
- Responsive to medication: Is the patient responsive to the medication, did the patient deteriorate after medication (yes/ no)?
- Cardiac surgery + date: Did the patient had any cardiac surgery (yes/ no). Which type of cardiac surgery did the patient had for the cardiac disease and on what date. Types of surgery that should be included e.g.:
  - Medical device: Pacemaker, ICD, CRT-P, CRT-D, LVAD
  - Heart transplantation/ cardiac reduction surgery
  - Angiography/ Stenting/ Angioplasty/ CABG
  - Valvular surgery
  - Pericardectomy
  - Ablation
  - Antiarrhythmic surgery (e.g. Maze)
  - Cardioversion
- Validation: Which method was used to validate the information about this cardiac event? If it is other please elaborate.
