# Supplementary material for: A New Method to Facilitate Valid and Consistent Grading Cardiac Events in Childhood Cancer Survivors Using Medical Records
Source: PLoS One. 2014 Jul 9;9(7):e100432. doi: 10.1371/journal.pone.0100432 (PMC4090125; doi:10.1371/journal.pone.0100432)
Supplement: File S1 — Manual text including background information on the different CEs, and an extensive explanation on the use of the method. (DOC) [file pone.0100432.s007.doc]

**Supportive Information S1:** Manual

Use of method

*What*

The following data is necessary for grading.

1. A specific description of the different symptoms of cardiac events, including (life threatening) symptoms

2. Information on additional diagnostic results (echocardiography,laboratory test , coronary angiography, blood etc.)

3. Information about cardiac medication and response to the medication

4. information about non-surgical treatment (Implantable cardioverter-defibrillator, Cardiac resynchronization therapy etc.)

5. Information about possible cardiac surgery

For the purpose of extracting this information, a data extraction form is developed (see Table S1).

*How*

The extra information for the first step of validation of the cases will be collected from one or more of the following:

• questionnaire/telephone to health care professional

• questionnaire/telephone to patient or their family, friends or social carer

• “late effects” clinic visit

• hospital discharge letter

• other

If the data is collected from a questionnaire, visit or letter, this should information should be validated with the medical chart or treating physician

*Grading*

The CTCAEv3.0 is used to grade the cardiac events, only grade 3, 4 and 5 are included. The flowcharts in Figure S1-S5 can be used to grade the cardiac events. For heart failure and ischemia we included life threatening consequences (grade 4), from CTCAEv4.0, this to make grading more simple/ consistent.

Heart failure (1, 2)

*Definition*

Heart failure is defined, clinically, as a syndrome in which patients have typical symptoms (e.g. breathlessness, ankle swelling, and fatigue) and signs (e.g. elevated jugular venous pressure, pulmonary crackles, and displaced apex beat) resulting from an abnormality of cardiac structure or function (Table S2-4).

*Grading*

If it is not known if there were any symptoms present/ related to the cardiac event, but an echocardiography was performed, and EF <40% or SF <15%, this cardiac event should be grade 3. If there is no echocardiography performed or there are no results, this cardiac event should not be included.

If the survivor is responsive to the given treatment? If so this cardiac event should be grade 3, if the survivor is not responsive to treatment, this cardiac event should be grade 4.

If it is not known, whether the survivor is responsive to the given treatment but there is evidence of a heart transplant or medical device this cardiac event should be grade 4, if not this cardiac event should be grade 3.

**Table S2:** Definition of heart failure

| **Heart failure is a clinical syndrome in which patients have the following features:** |
| --- |
| **Symptoms typical of heart failure** (breathlessness at rest or on exercise, fatigue, tiredness, ankle swelling)  AND |
| **Signs typical of heart failure** (tachycardia, tachypnoea, pulmonary rales, pleural effusion, raised jugular venous pressure, peripheral oedema, hepatomegaly)  AND |
| **Objective evidence of a structural or functional abnormality of the heart at rest** (cardiomegaly, third heart sound, cardiac murnurs, abnormality on the echocardiogram, raised natriuretic peptide concentration) |

**Table S3:** Common clinical manifestation of heart failure

| **Dominant clinical feature** | **Symptoms** | **Signs** |
| --- | --- | --- |
| Peripheral oedema/ congestion | - Breathlessness - Tiredness, Fatigue - Anorexia | - Peripheral oedema - Raised jugular venous pressure - Pulmonary oedema - Hepatomegaly, ascites - Fluid overload (congestion) - Cachexia |
| Pulmonary oedema | - Severe breathlessness at rest | - Crackles or rales over lungs, effusion - tachycardia, tachypnoea |
| Cardiogenic shock (low output syndrome | - Confusion - Weakness - Cold | - Poor peripheral perfusion - SBP <90 mmHg - Anuria or oliguria |
| High blood pressure (hypertensive heart failure) | - Breathlessness | - Usually raised blood pressure, left ventricular hypertrophy and preserved ejection fraction |
| Right heart failure | - Breathlessness - Fatigue | - Evidence of right ventricular dysfunction - Raised jugular venous pressure, peripheral oedema, hepatomegaly, gut congestion |

**Table S4:** New York Heart Association functional classification based on severity of symptoms and physical activity

| **Class I** | No limitations of physical activity. Ordinary physical activity does not cause undue breathlessness, fatigue or palpitations |
| --- | --- |
| **Class II** | Slight limitations of physical activity. Comfortable at rest, but ordinary physical activity result in undue breathlessness, fatigue or palpitations |
| **Class III** | Marked limitations of physical activity. Comfortable at rest, but less than ordinary physical activity result in undue breathlessness, fatigue or palpitations |
| **Class IV** | Unable to carry on any physical activity without discomfort. Symptoms at rest can be present. If any physical activity is undertaken, discomfort is increaded. |

Ischemia (3, 4)

*Definition*

The clinical presentations of coronary artery disease (CAD) include silent ischaemia, stable angina pectoris, unstable angina, myocardial infarction (MI), heart failure, and sudden death. In this study only unstable angina and myocardial infarction are included. The main symptom of CAD is the chest pain.

*Grading*

If it is not known whether the event was symptomatic or if the symptoms were related to the cardiac event, or if the event was symptomatic, but testing was not consistent with ischemia or whether it was unknown, this cardiac event should not be included (≤grade 2/ unknown).

If testing was consistent with ischemia, but was not a myocardial infarction or if the event had not any life threatening consequences this cardiac event should be grade 3, if the event was a myocardial infarction or if the event had any life threatening consequences this cardiac event should be grade 4.

Pericarditis (5, 6)

*Grading*

Pericarditis with only symptoms (e.g. chest pain), without physiological consequences like pericardial constriction/ effusion is a grade 2 event, and should not be included.

If the survivor also has physiological consequences like pericardial constriction and pericardial effusion and no life threatening consequences or it is unknown whether there were life threatening consequences, this cardiac event should be grade 3.

If there were life threatening consequences, this cardiac event should be grade 4.

Valvular disease (7)

*Grading*

Only events with symptoms of severe regurgitation or stenosis and controlled with interventions should be grade 3. If it is unknown whether the survivor had symptoms of severe reguration or stenosis or if the symptoms were related to the cardiac event, but is controlled with an intervention, this cardiac event should be grade 3.If the valvular disease is not controlled with intervention or if it is unknown but the survivor needs a valve replacement or valvuloplasty or has life threatening symptoms this cardiac event should be grade 4.If it is known that the survivor had symptoms, but it is unknown whether the valvular disease was controlled with interventions and it is unknown whether the survivor had a valve replacement or valvuloplasty or had life threatening symptoms this cardiac event should be grade 3.

Arrhythmia (8, 9)

*Grading*

Arrhythmia events are a grade 3 event, if they are controlled with medicine, surgery or a device (including cardioversion). If not, this cardiac event should not be included (≤grade 2). To clarify; symptomatic events, but without medication, surgery or a device should be a grade 2 event, thus not an inclusion.

If there were any life threatening consequences of the arrhythmia, this cardiac event should be grade 4.

**References**

1. Dickstein K, Cohen-Solal A, Filippatos G, McMurray JJV, Ponikowski P et al. (2008) ESC Guidelines for the diagnosis and treatment of acute and chronic heart failure 2008. The Task Force for the Diagnosis and Treatment of Acute and Chronic Heart Failure 2008 of the European Society of Cardiology. Developed in collaboration with the Heart Failure Association (HFA) of the ESC and endorsed by the ESICM. Eur Heart J 29:2388-442.
2. McMurray JJV, Adamopoulos S, Anker SD, Auricchio A, Bohm M et al. (2012) ESC Guidelines for the diagnosis and treatment of acute and chronic heart failure 2012. The Task Force for the Diagnosis and Treatment of Acute and Chronic Heart Failure 2012 of the European Society of Cardiology Developed in collaboration with the Heart Failure Association (HFA) of the ESC.. Eur Heart J doi:10.1093/eurheartj/ehs104.
3. Thygesen K, Alpert JS and White HD on behalf of the jount ESC/ American college of cardiology foundation (ACCF)/ American Heart Association (AHA) (2007) WHF Task force for the redefinition of myocardial infarction. Universal definition of myocardial infarction. Eur Heart J 28:2525-38.
4. Van der Werf F, Bax J, Betriu A, Blomström-Lundqvist C, Crea F et al. (2009) Management of acute myocardial infarction in patients presenting with persistent ST-segment elevation. The Task Force on the management of ST-segment elevation acute myocardial infarction of the European Society of Cardiology. Eur Heart J 29:2909-45.
5. Maisch B, Seferovic PM, Ristic AD, Erbel R, Rienmuller R et al. (2004) Guidelines on the Diagnosis and Management of Pericardial Diseases. The Task Force on the Diagnosis and Management of Pericardial Diseases of the European Society of Cardiology. Eur Heart J 25(7):1–28
6. Kumar PP. (1980) Pericardial injury from mediastinal irradiation. J Nat Med Ass 72(6):591-4
7. Bonow RO, Carabello BA, Chatterjee K, de Leon AC, Faxon DP, Freed MD et al. (2006) ACC/AHA 2006 Guidelines for the Management of Patients With Valvular Heart Disease. Circ 114:e84-e231.
8. Blomström-Lundqvist C, Scheinman MM, Aliot EM, Alpert JS, Calkins H et al. (2003) ACC/AHA/ESC guidelines for the management of patients with supraventricular arrhythmias: a report of the American College of Cardiology/American Heart Association Task Force on Practice Guidelines and the European Society of Cardiology Committee for Practice Guidelines (Writing Committee to Develop Guidelines for the Management of Patients With Supraventricular Arrhythmias. American College of Cardiology Web Site. Available at: http://www.cardiosource.org/~/media/Images/ACC/Science%20and%20Quality/Practice%20Guidelines/s/sva_index.ashx
9. Zipes DP, Camm AJ, Borgette M. Buxton AE, Chaitman B et al. (2006) ACC/ AHA/ ASC 2006 guidelines for management of patients with vertricular arrhythmias and the prevention of sudden cardiac death A Report of the American College of Cardiology/American Heart Association Task Force and the European Society of Cardiology Committee for Practice Guidelines (Writing Committee to Develop Guidelines for the Management of Patients With Ventricular Arrhythmias and the Prevention of Sudden Cardiac Death). Europace 8:746-837.
