# Supplementary material for: A New Method to Facilitate Valid and Consistent Grading Cardiac Events in Childhood Cancer Survivors Using Medical Records
Source: PLoS One. 2014 Jul 9;9(7):e100432. doi: 10.1371/journal.pone.0100432 (PMC4090125; doi:10.1371/journal.pone.0100432)
Supplement: Presentation S1 — Training presentation. (PPS) [file pone.0100432.s008.pps]

## Slide 1
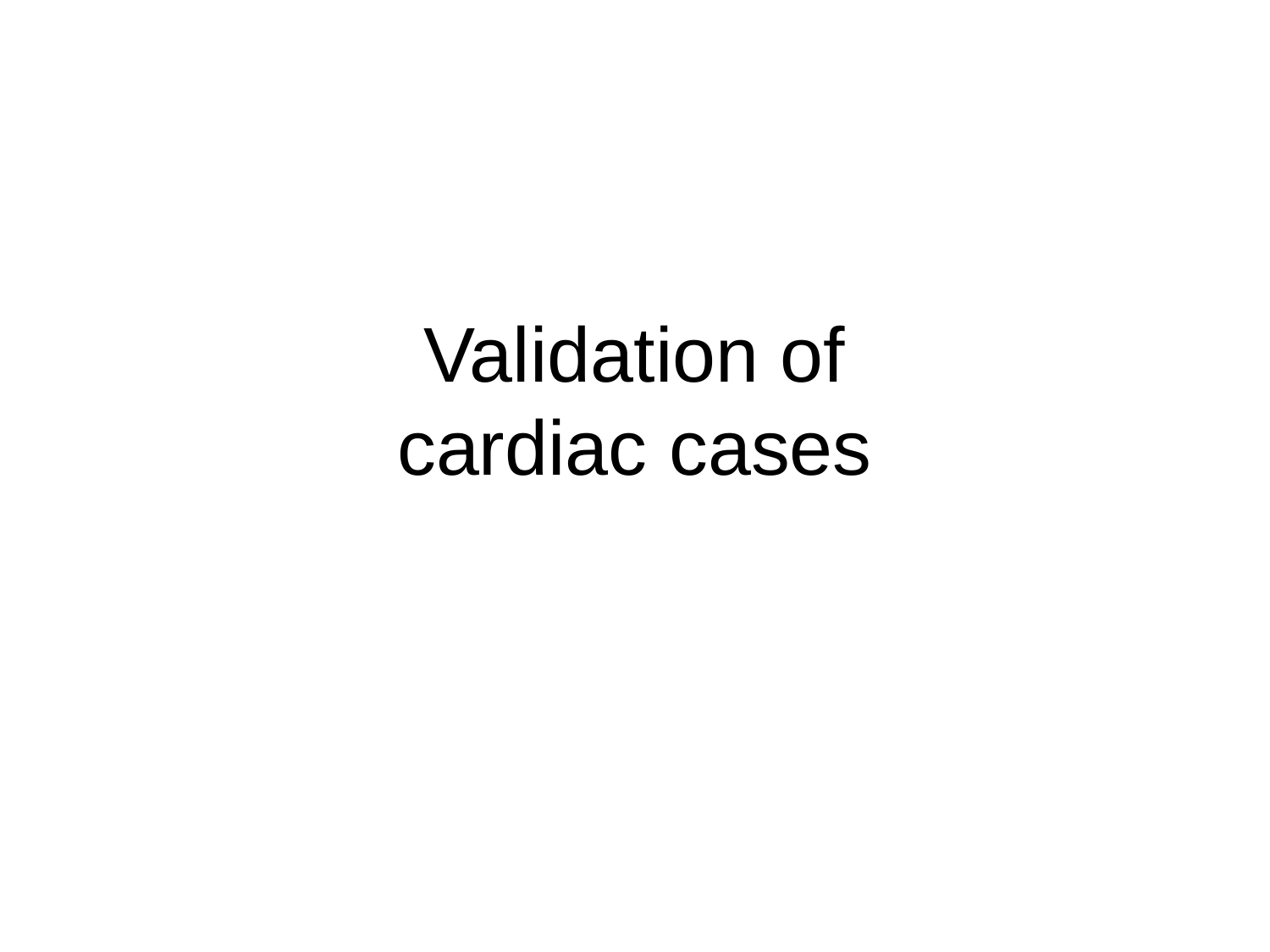

# Validation of cardiac cases

## Slide 2
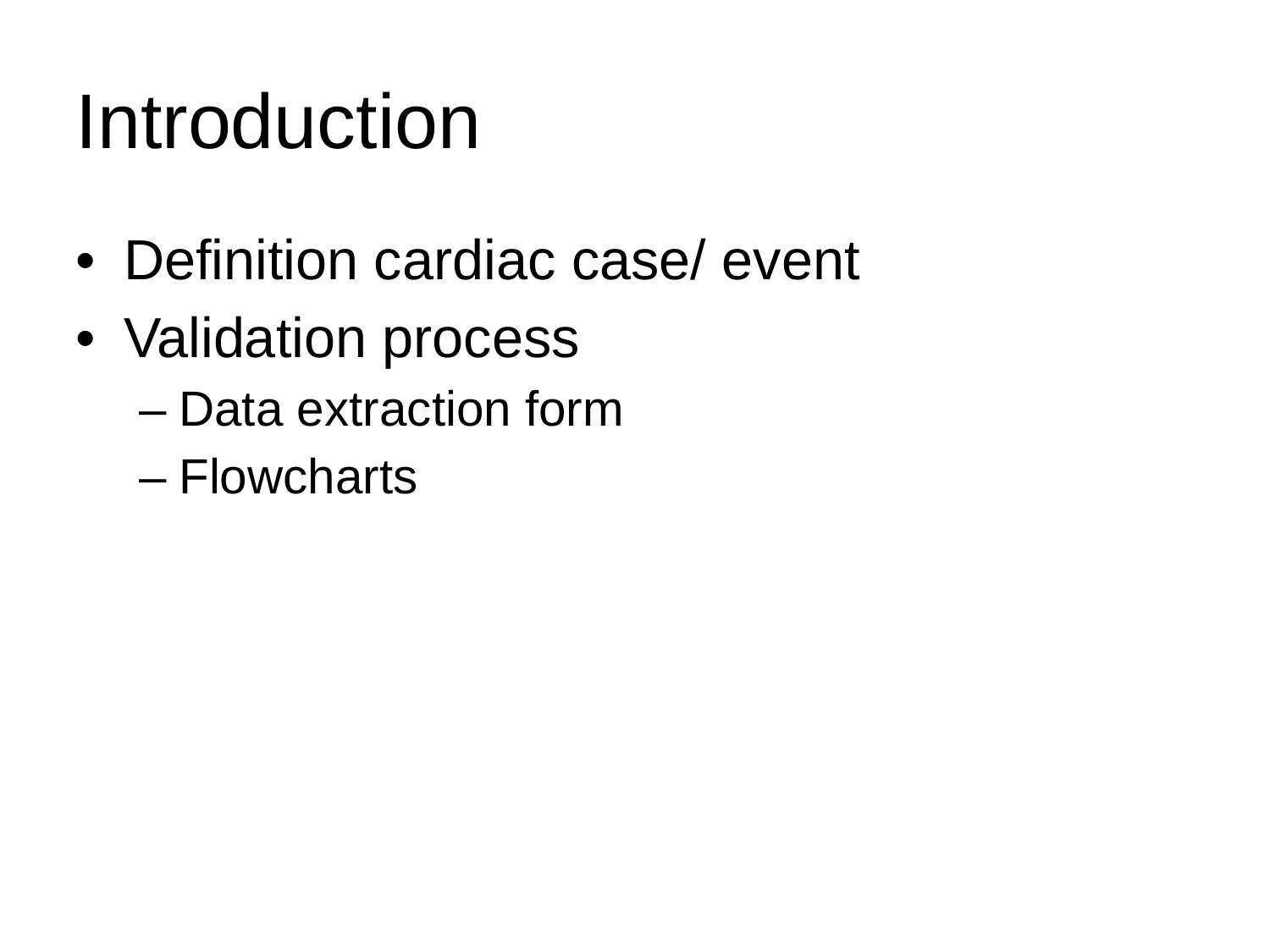

Introduction
# Definition cardiac case/ event
Validation process
Data extraction form
Flowcharts

## Slide 3
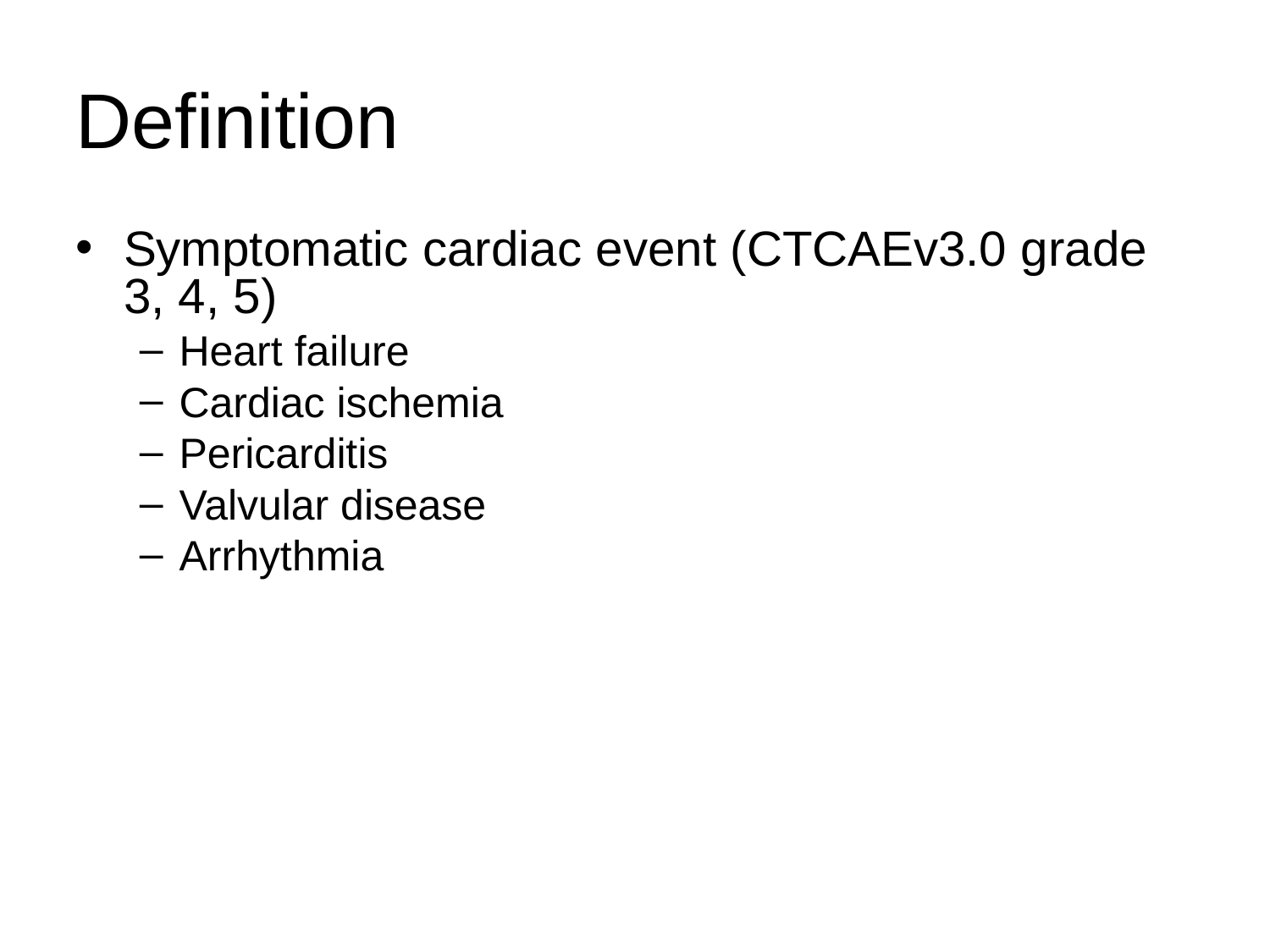

Definition
# Symptomatic cardiac event (CTCAEv3.0 grade 3, 4, 5)
Heart failure
Cardiac ischemia
Pericarditis
Valvular disease
Arrhythmia

## Slide 4
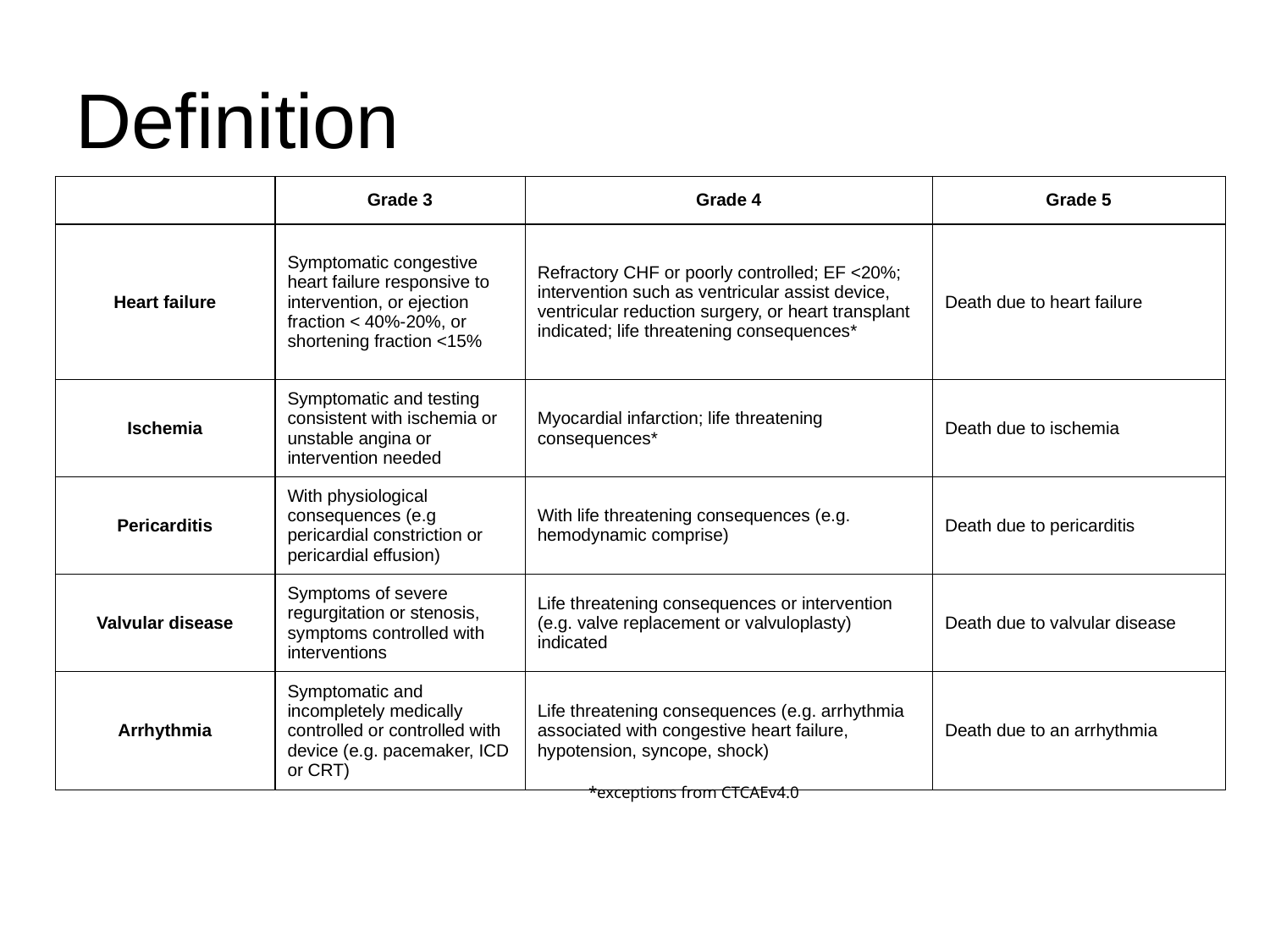

# Definition
| | Grade 3 | Grade 4 | Grade 5 |
| --- | --- | --- | --- |
| Heart failure | Symptomatic congestive heart failure responsive to intervention, or ejection fraction < 40%-20%, or shortening fraction <15% | Refractory CHF or poorly controlled; EF <20%; intervention such as ventricular assist device, ventricular reduction surgery, or heart transplant indicated; life threatening consequences\* | Death due to heart failure |
| Ischemia | Symptomatic and testing consistent with ischemia or unstable angina or intervention needed | Myocardial infarction; life threatening consequences\* | Death due to ischemia |
| Pericarditis | With physiological consequences (e.g pericardial constriction or pericardial effusion) | With life threatening consequences (e.g. hemodynamic comprise) | Death due to pericarditis |
| Valvular disease | Symptoms of severe regurgitation or stenosis, symptoms controlled with interventions | Life threatening consequences or intervention (e.g. valve replacement or valvuloplasty) indicated | Death due to valvular disease |
| Arrhythmia | Symptomatic and incompletely medically controlled or controlled with device (e.g. pacemaker, ICD or CRT) | Life threatening consequences (e.g. arrhythmia associated with congestive heart failure, hypotension, syncope, shock) | Death due to an arrhythmia |
*exceptions from CTCAEv4.0

## Slide 5
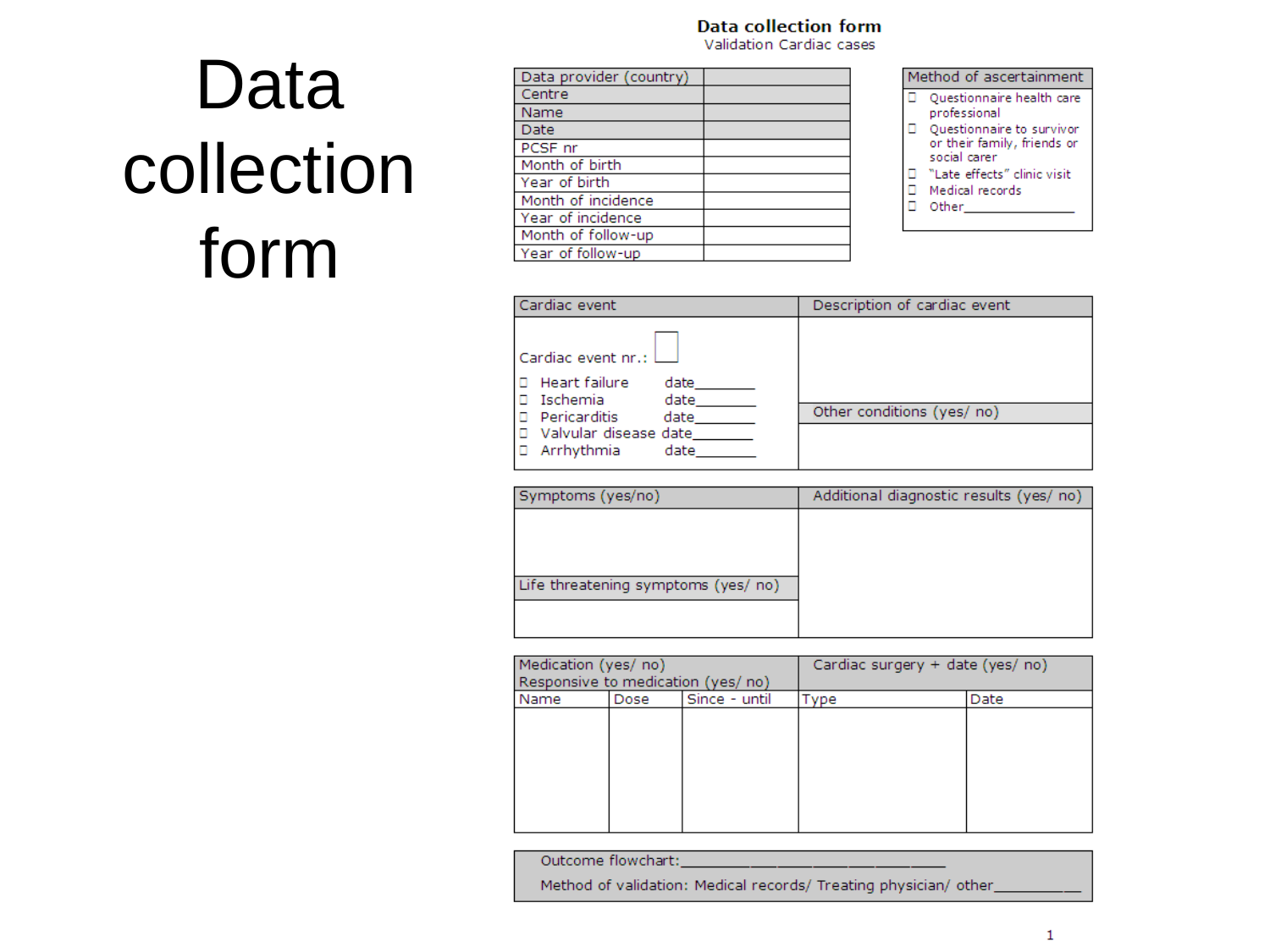

# Data collection form

## Slide 6
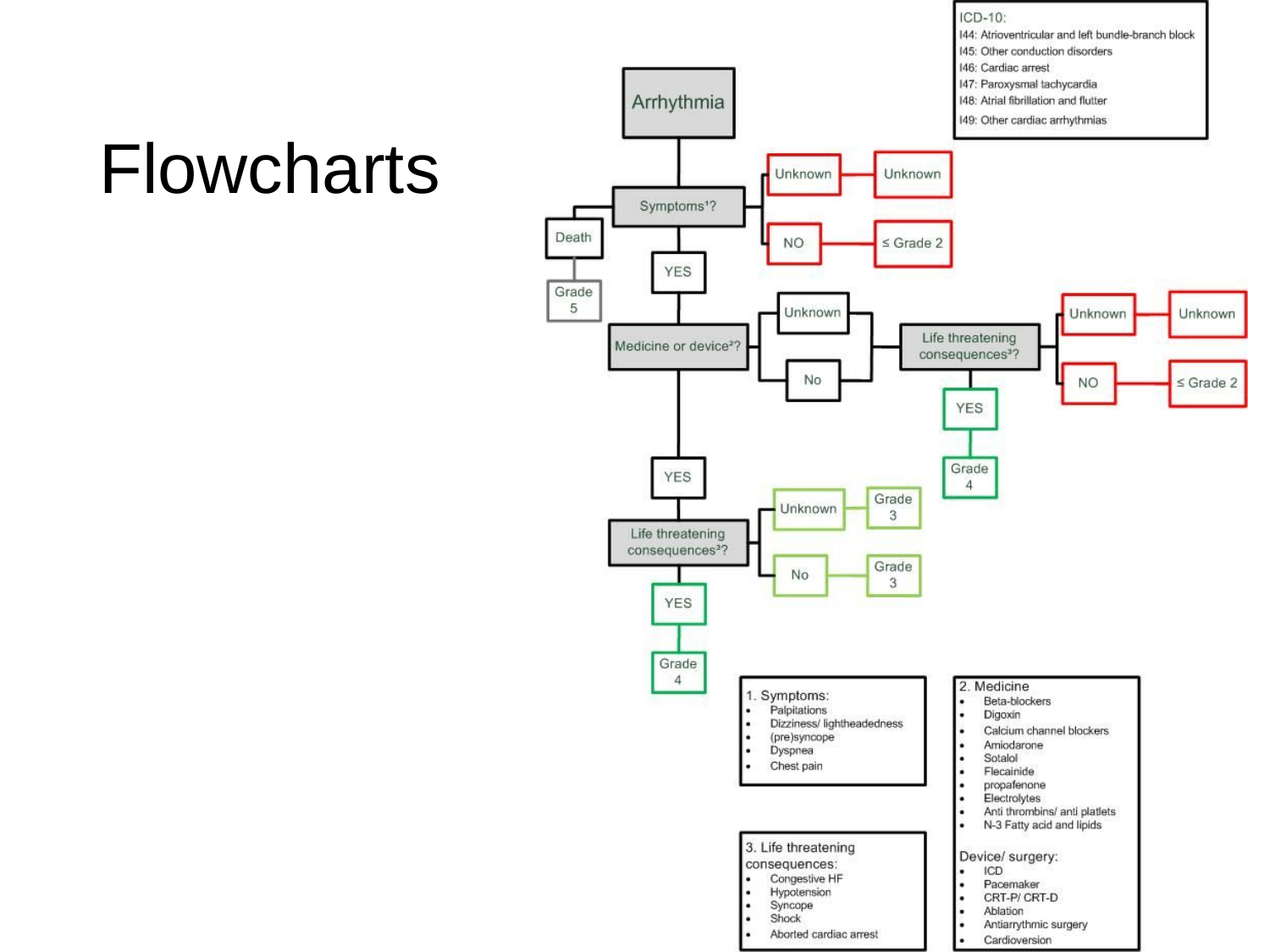

Flowcharts

## Slide 7
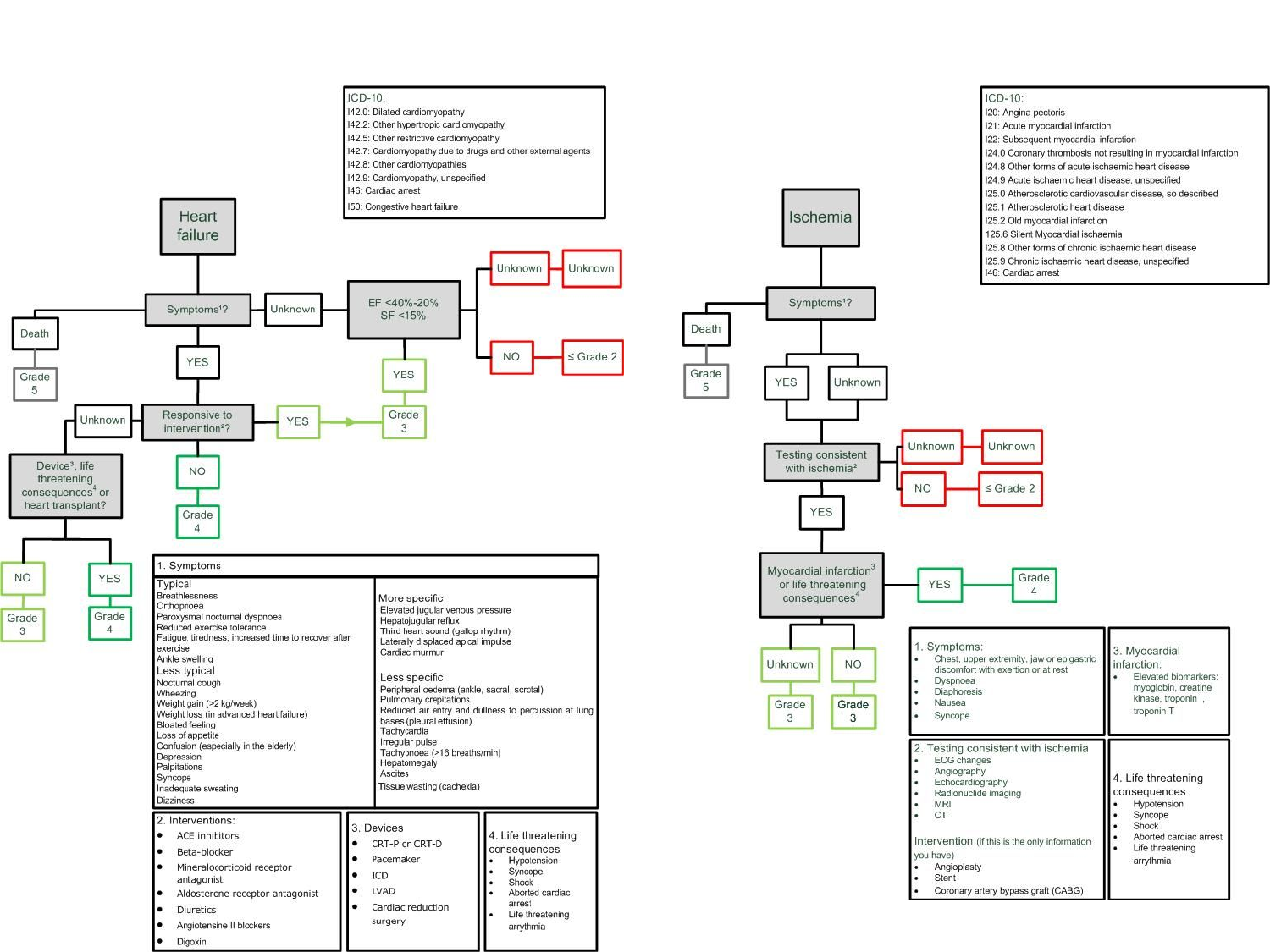

## Slide 8
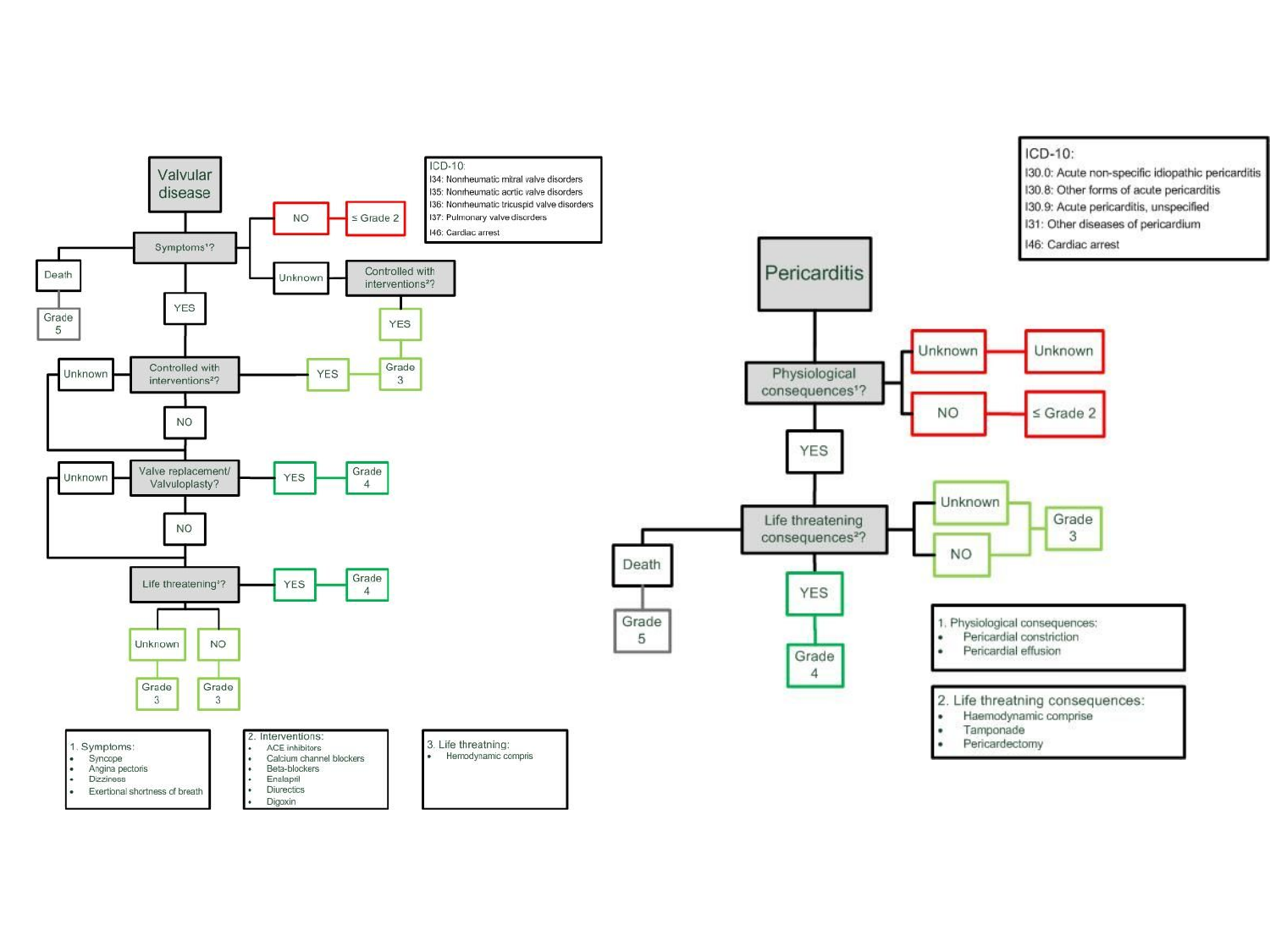

## Slide 9
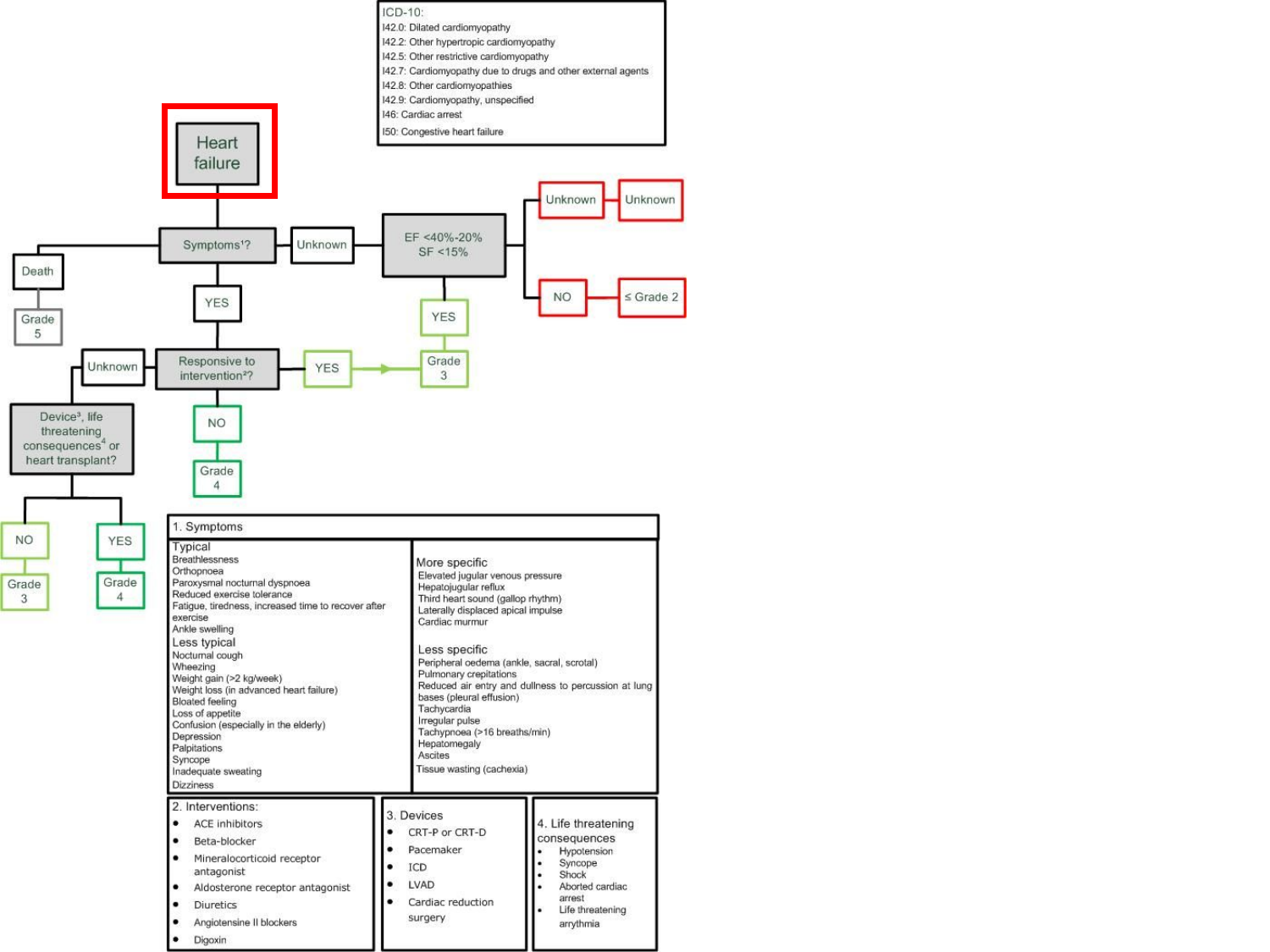

## Slide 10
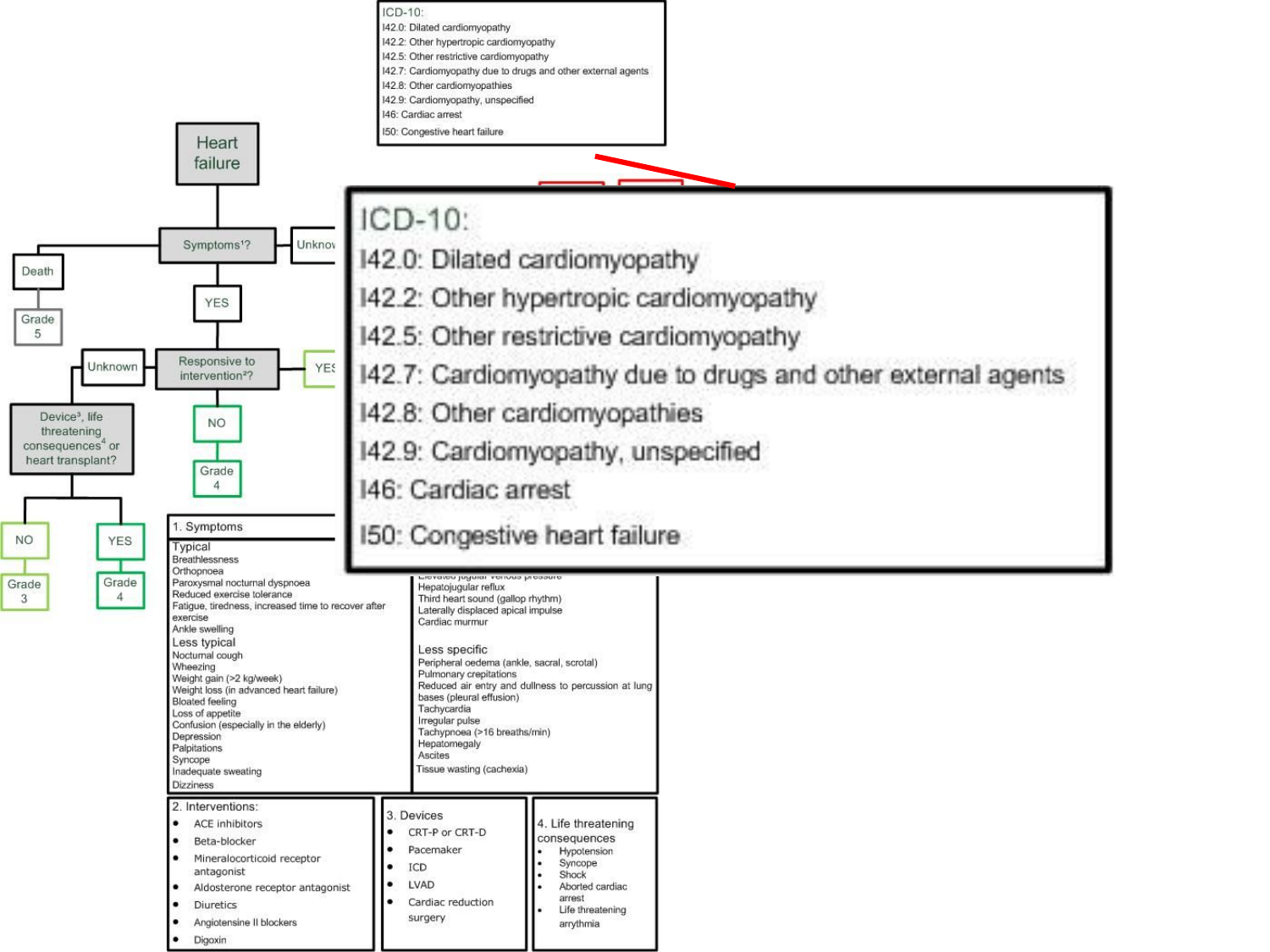

## Slide 11
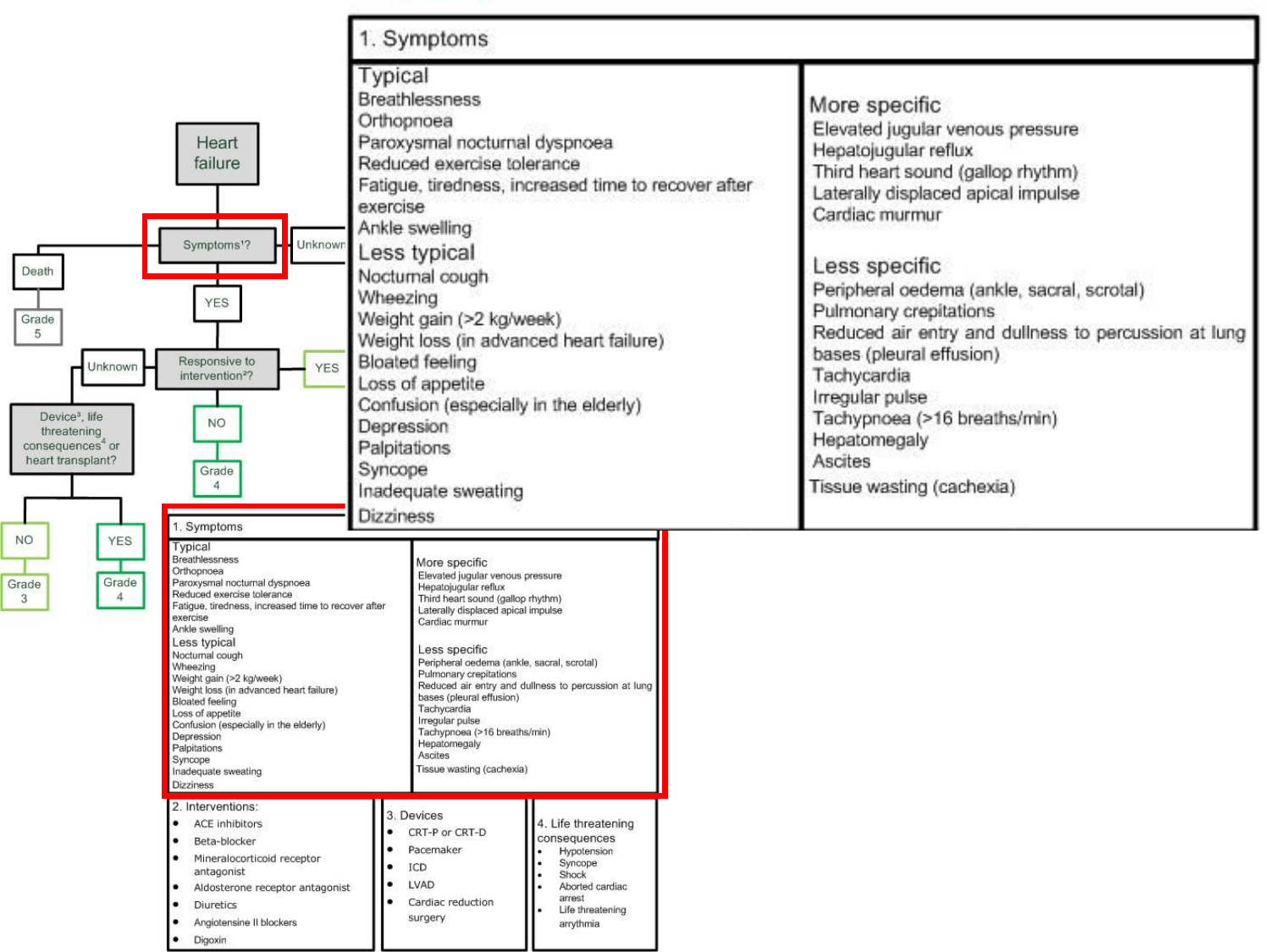

## Slide 12
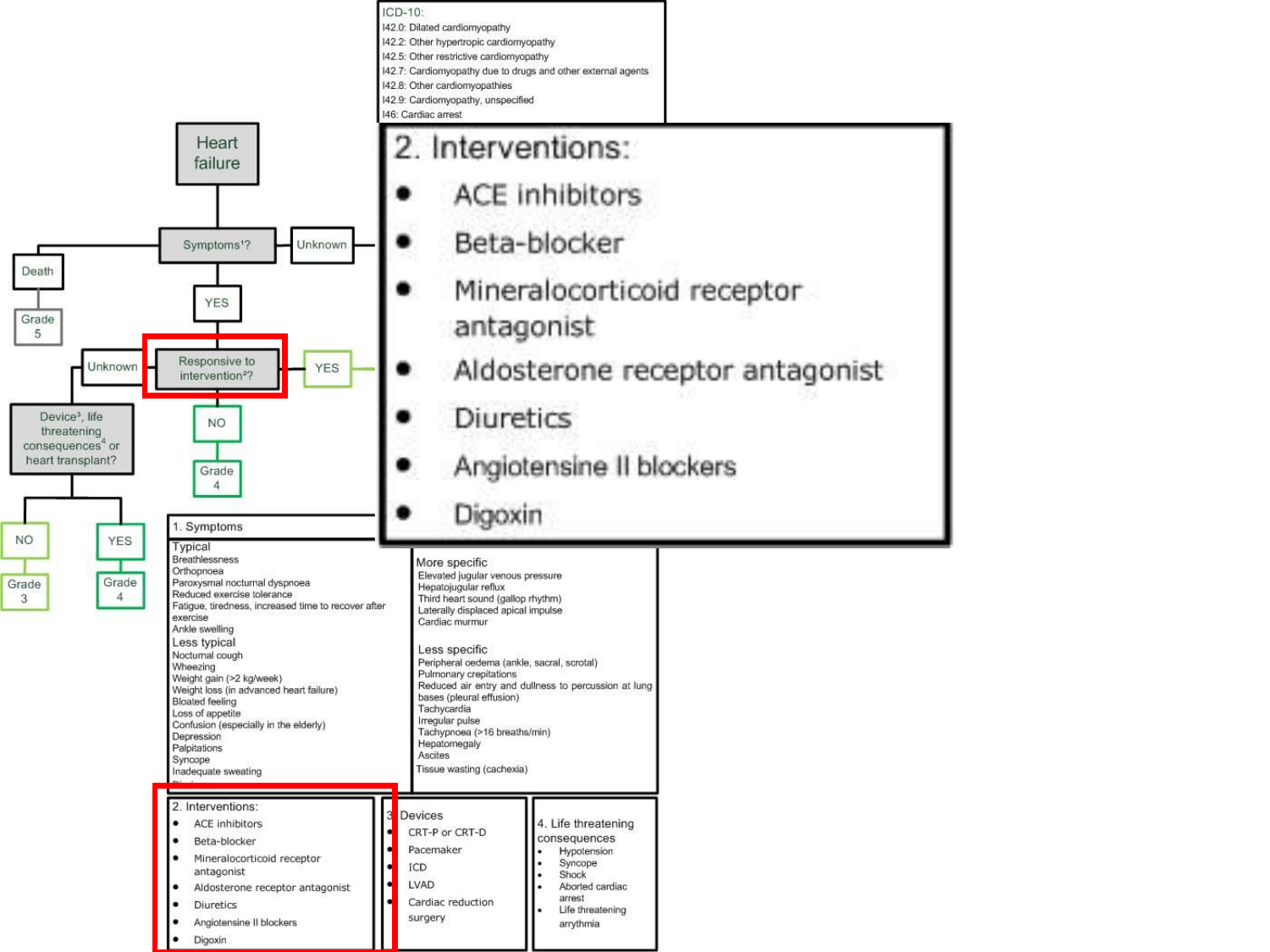

## Slide 13
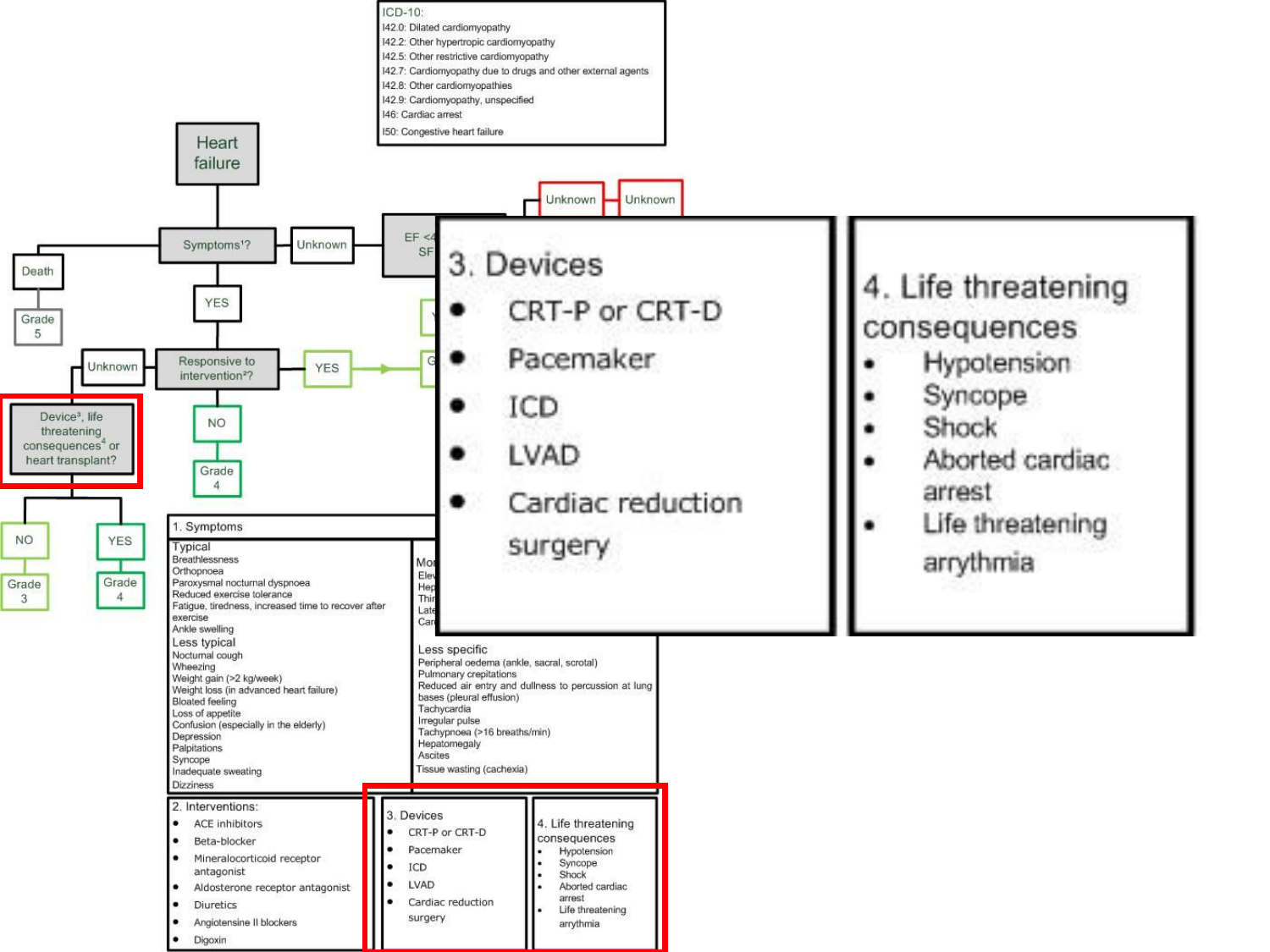

## Slide 14
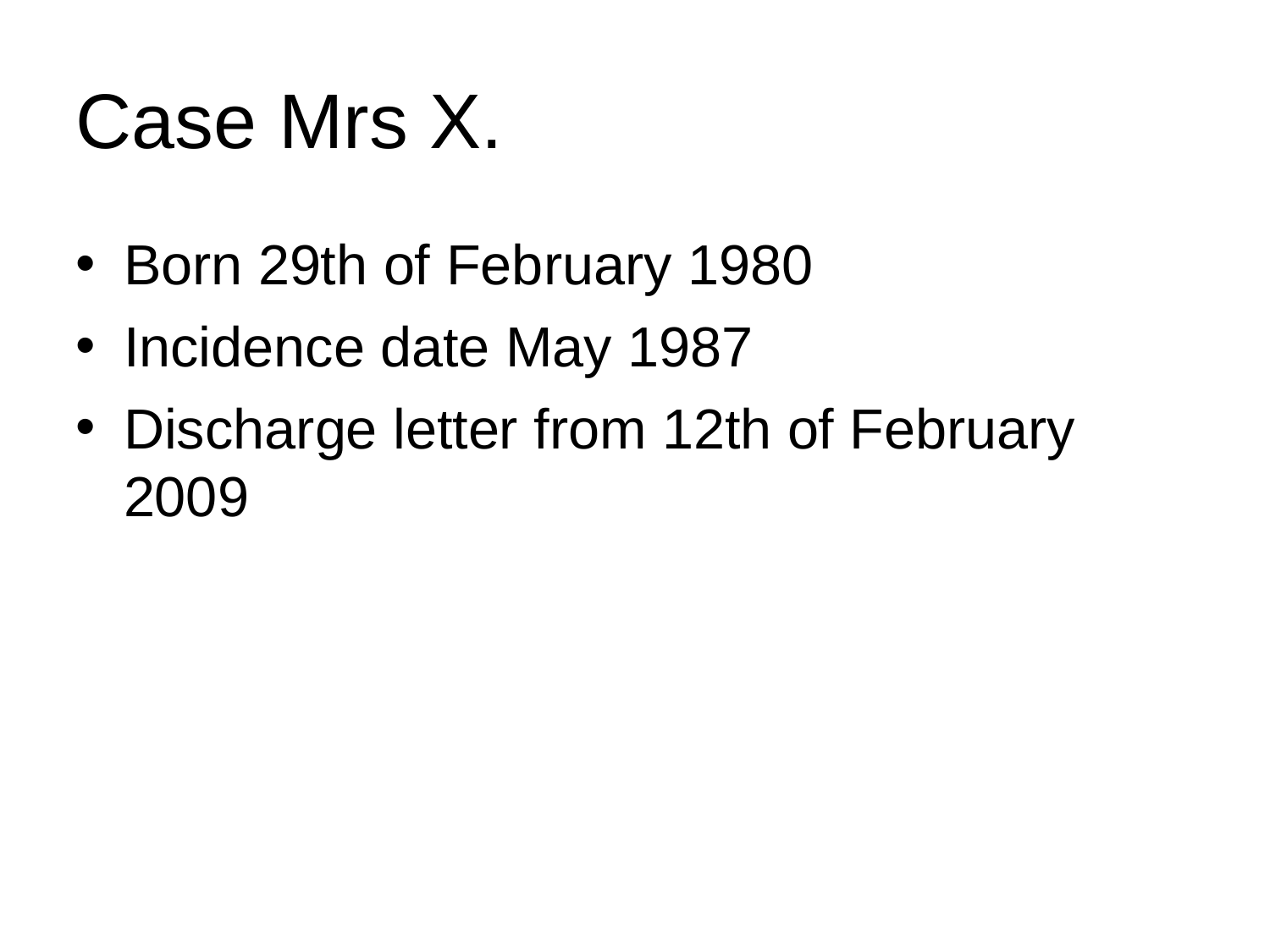

Case Mrs X.
# Born 29th of February 1980
Incidence date May 1987
Discharge letter from 12th of February 2009

## Slide 15
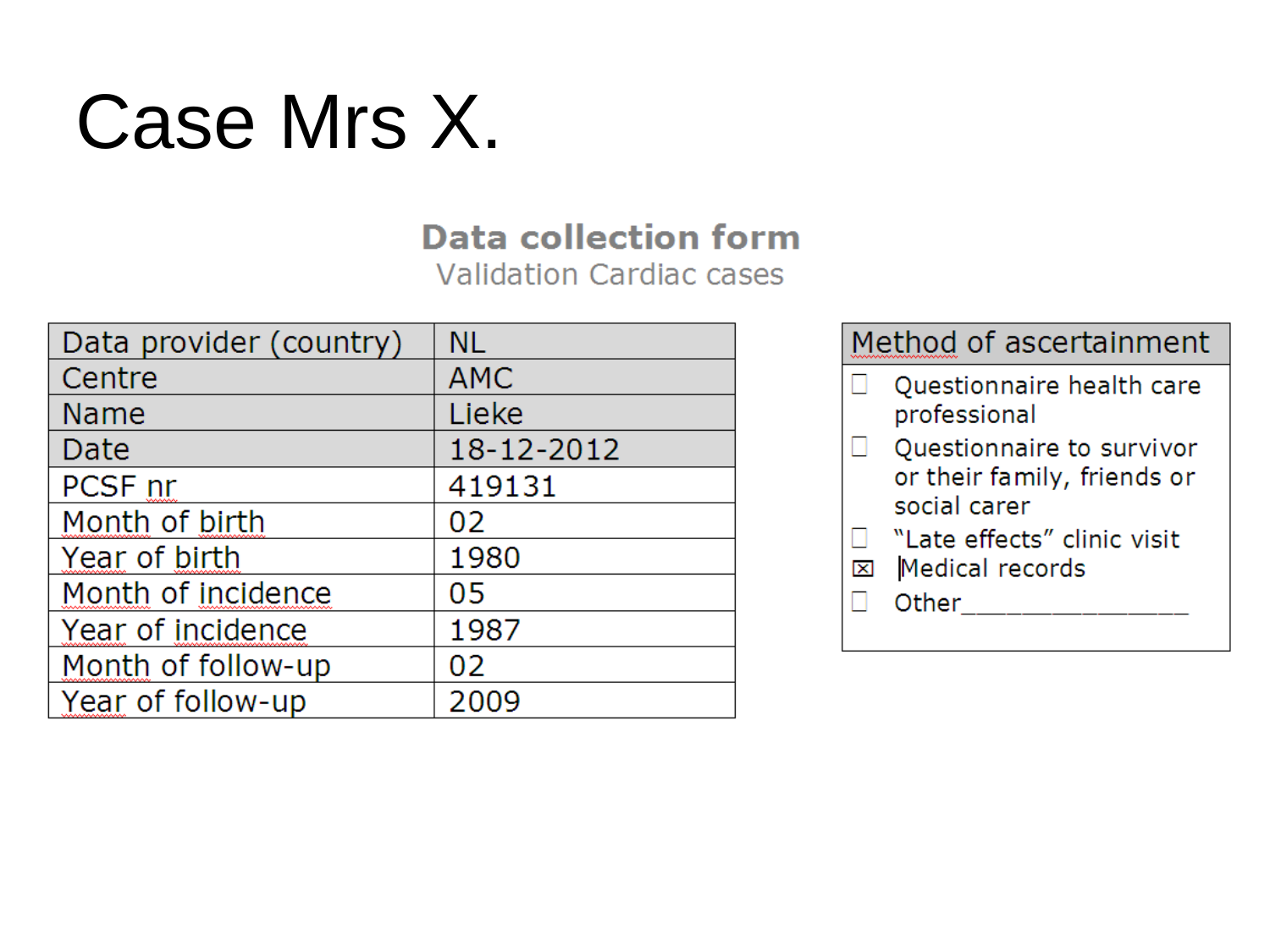

# Case Mrs X.

## Slide 16
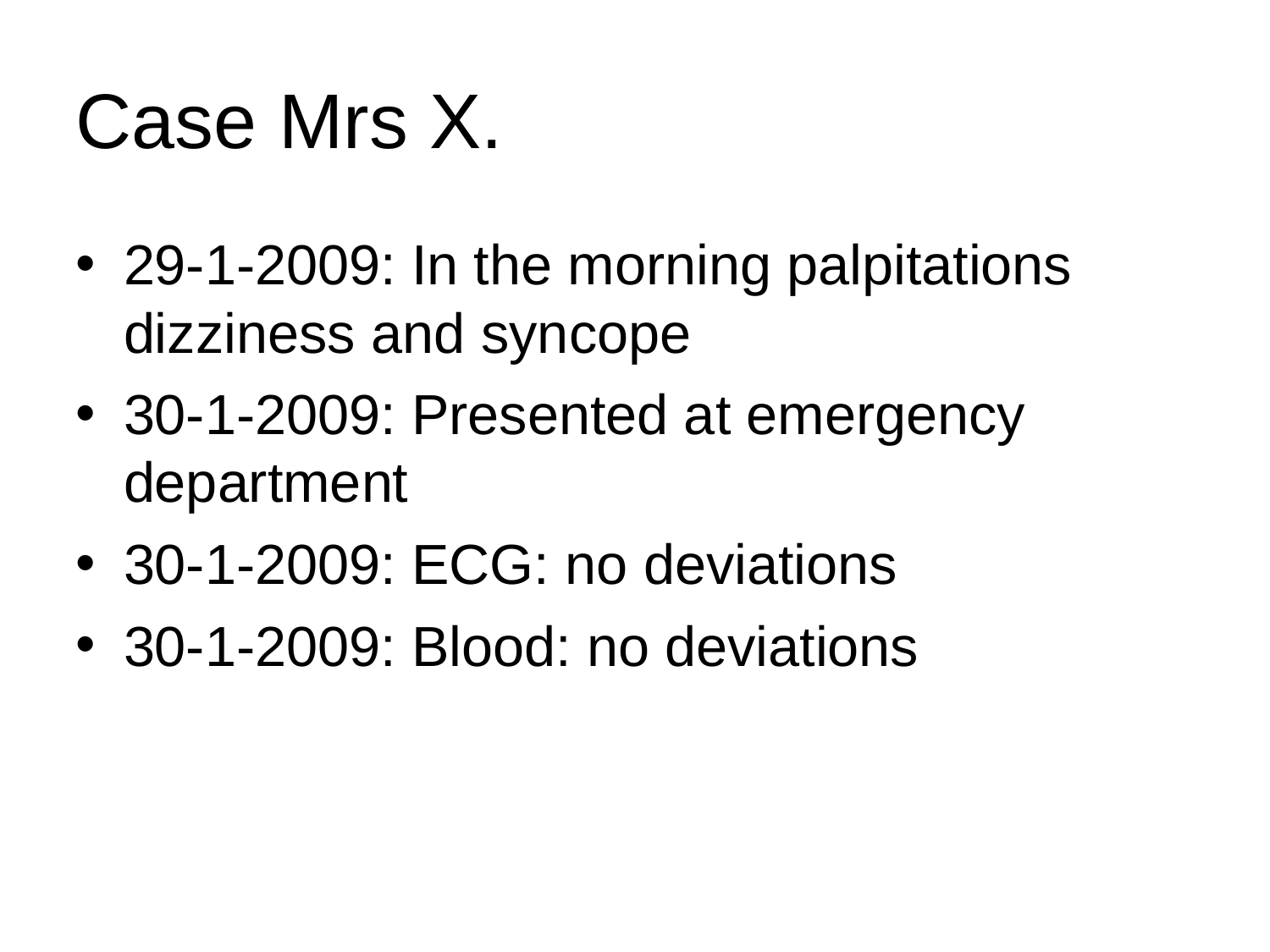

Case Mrs X.
# 29-1-2009: In the morning palpitations dizziness and syncope
30-1-2009: Presented at emergency department
30-1-2009: ECG: no deviations
30-1-2009: Blood: no deviations

## Slide 17
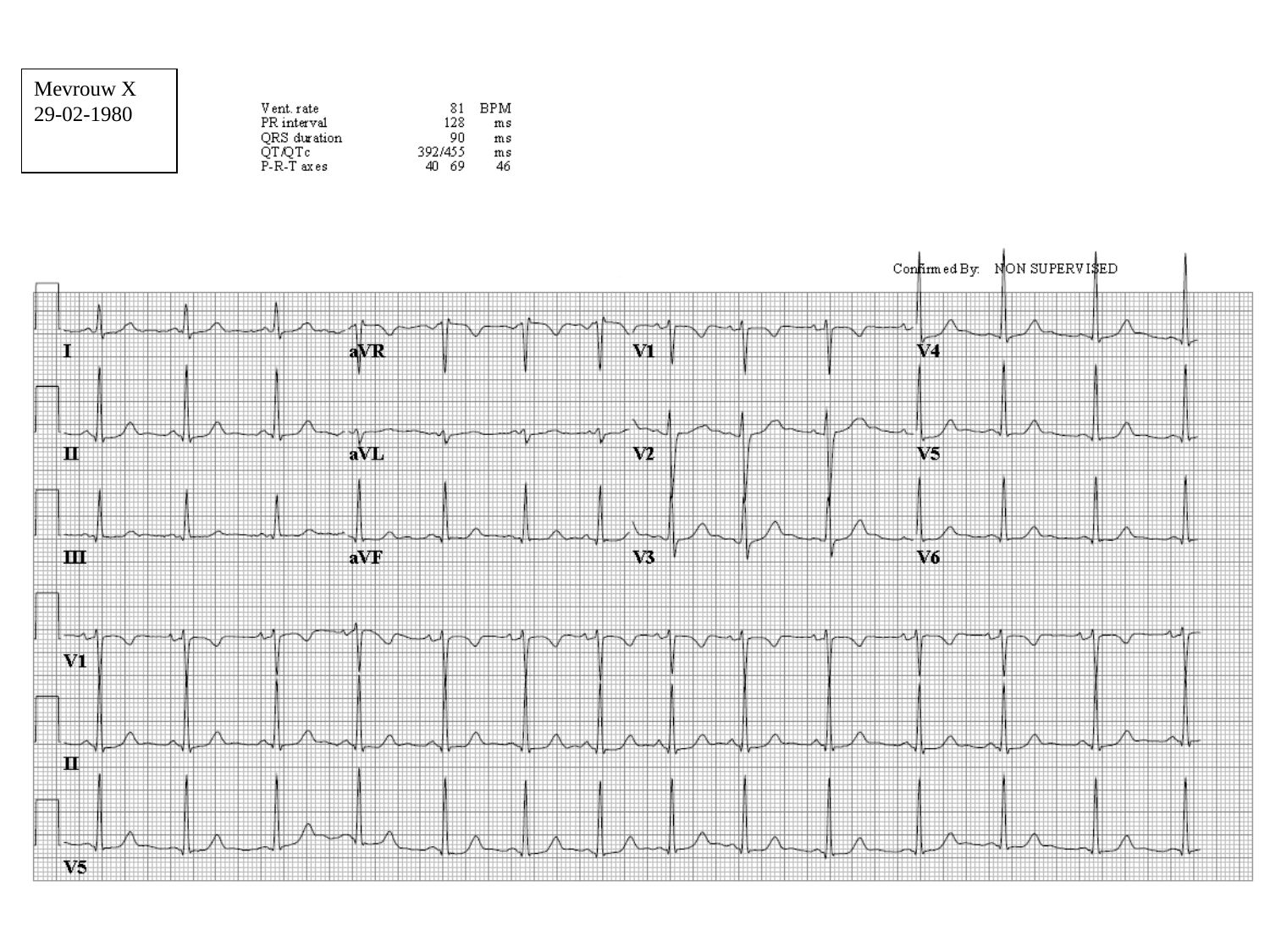

Mevrouw X
29-02-1980

## Slide 18
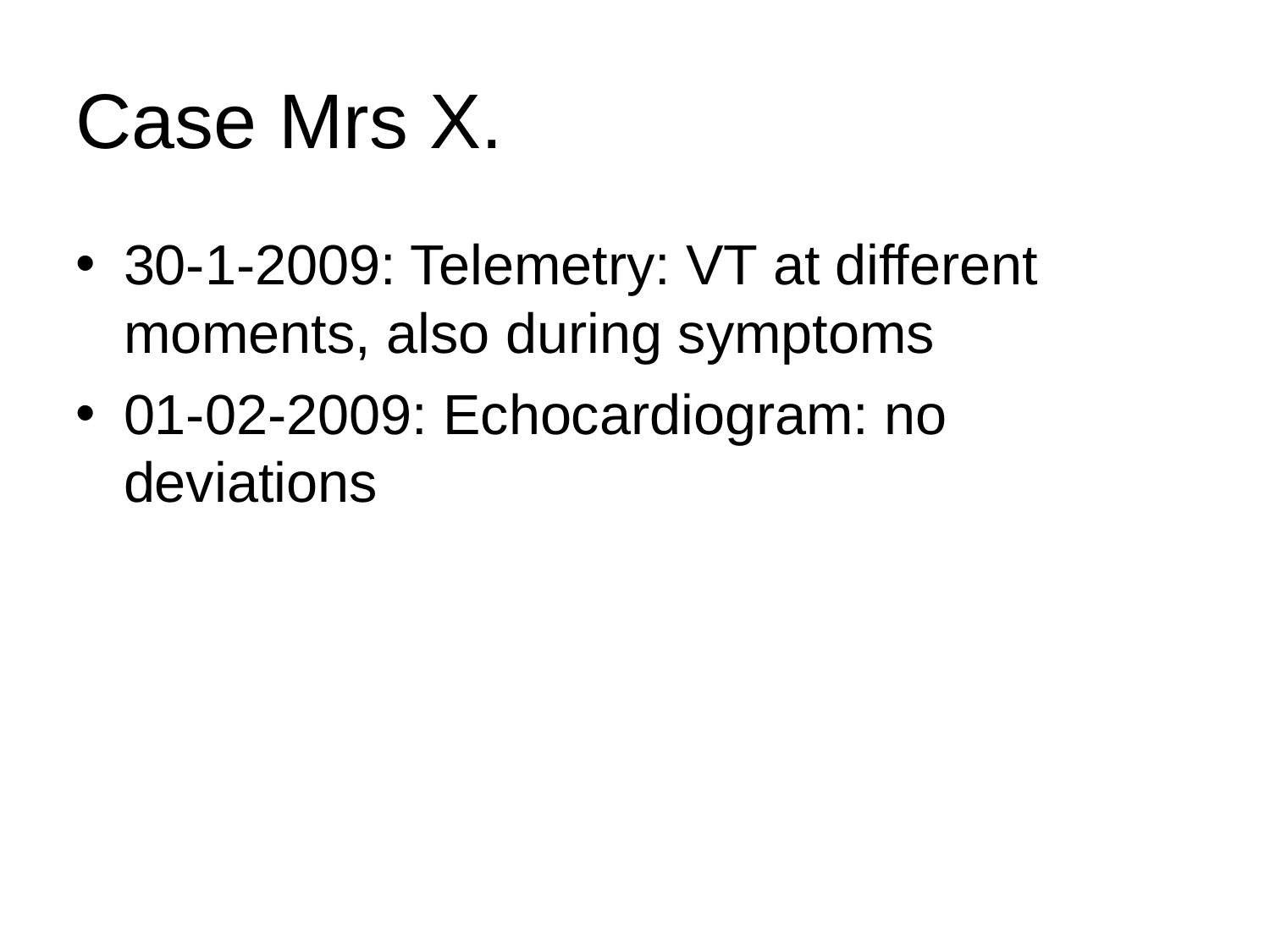

Case Mrs X.
# 30-1-2009: Telemetry: VT at different moments, also during symptoms
01-02-2009: Echocardiogram: no deviations

## Slide 19
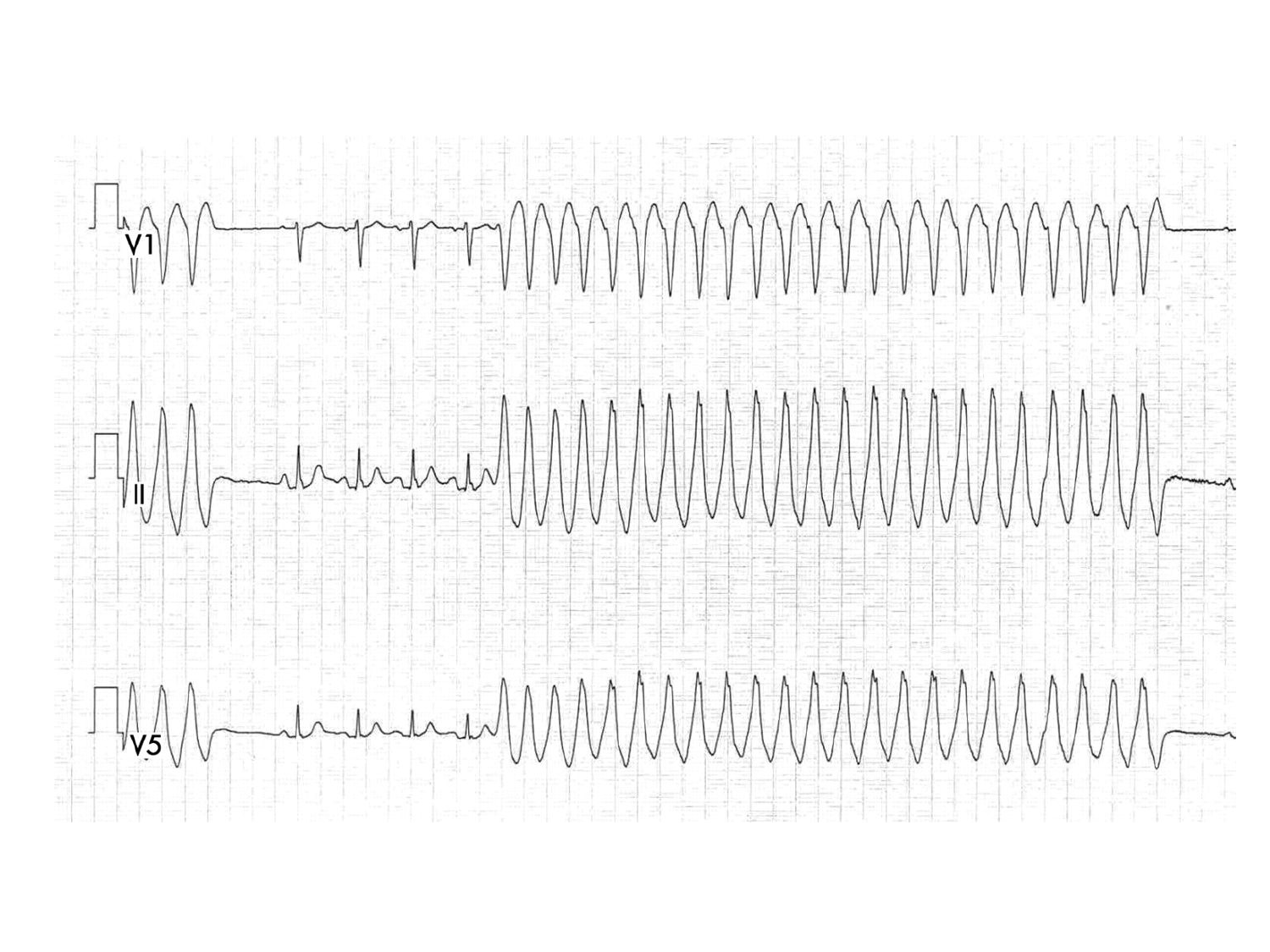

## Slide 20
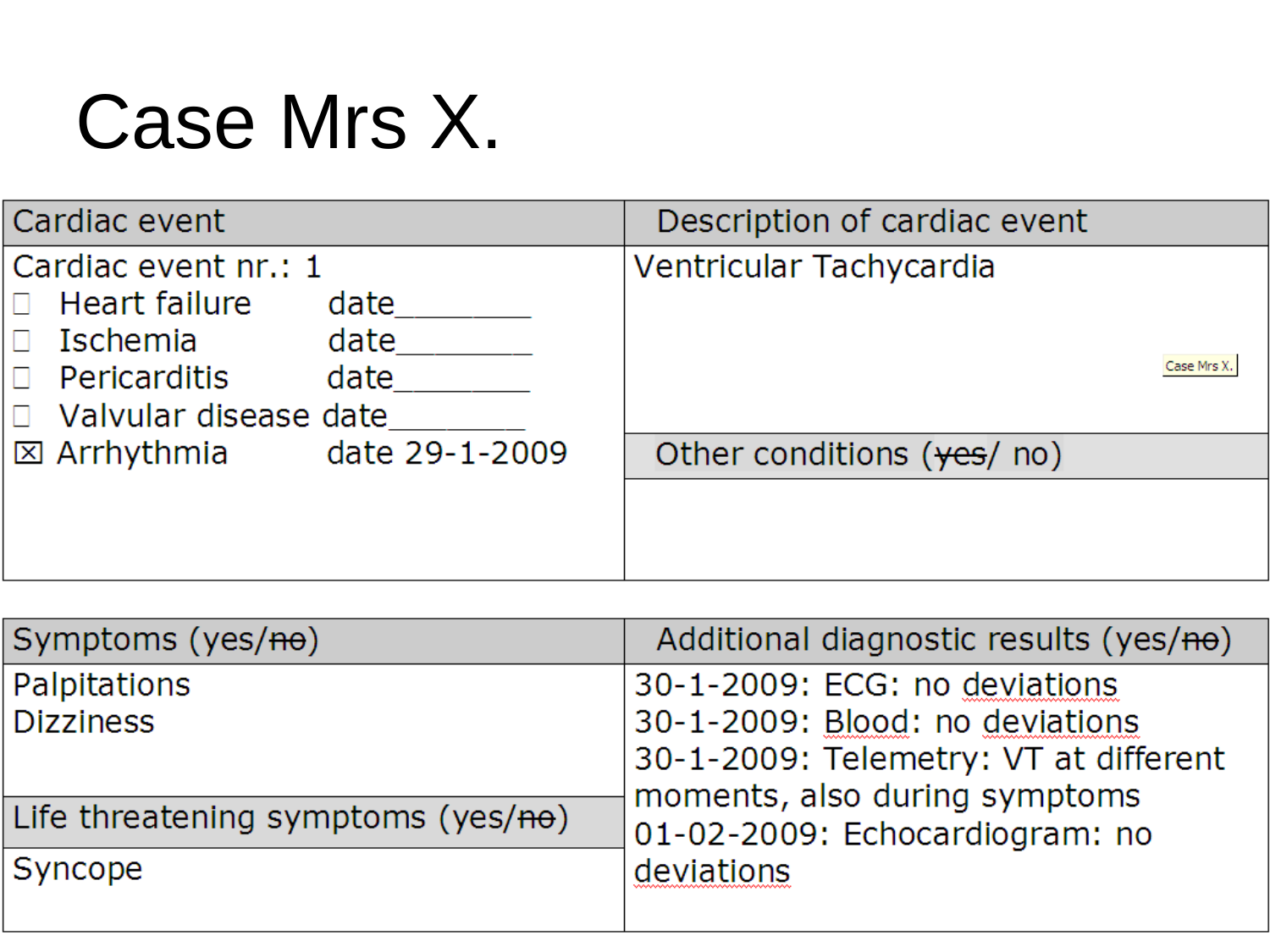

# Case Mrs X.

## Slide 21
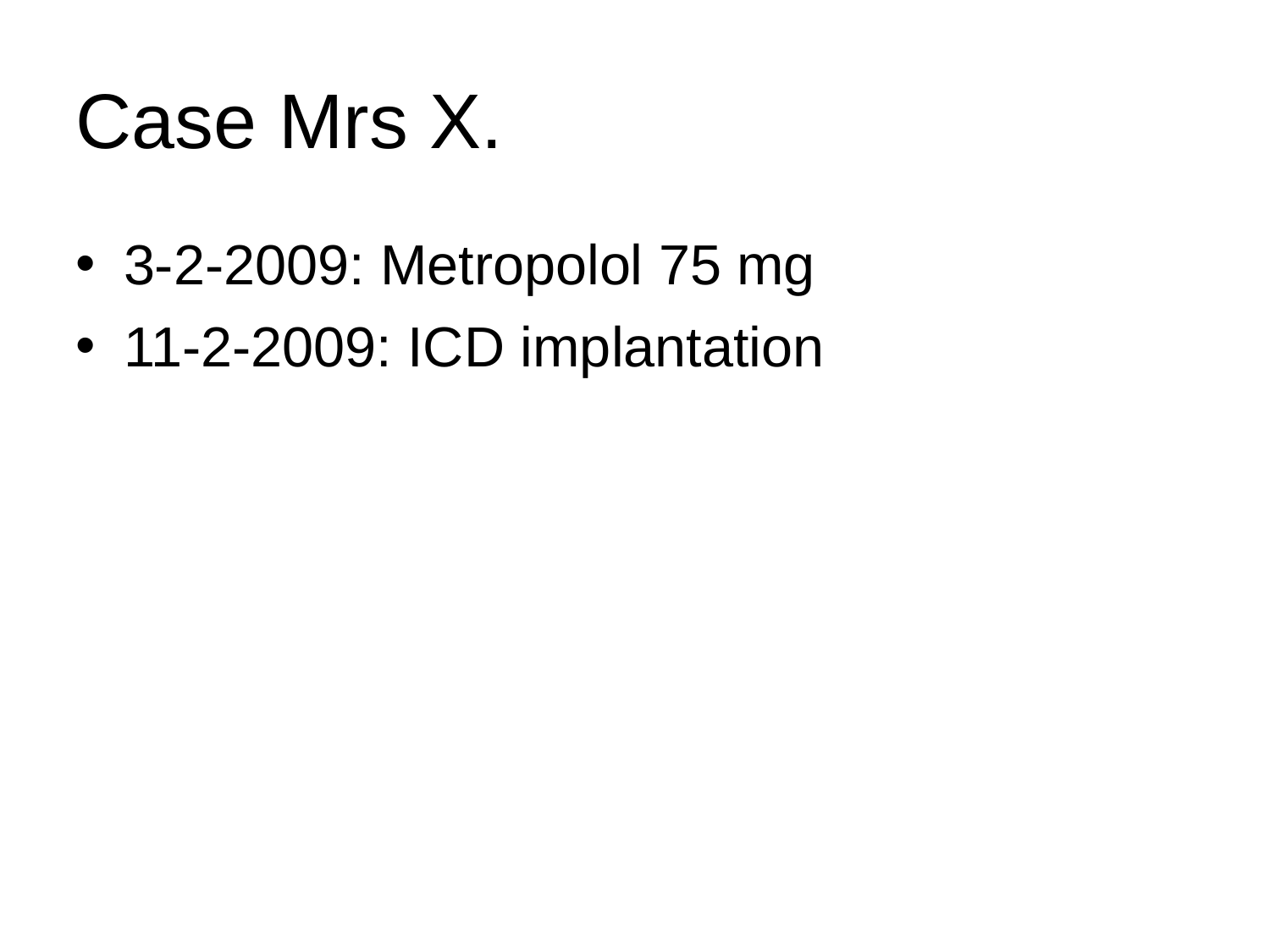

Case Mrs X.
# 3-2-2009: Metropolol 75 mg
11-2-2009: ICD implantation

## Slide 22
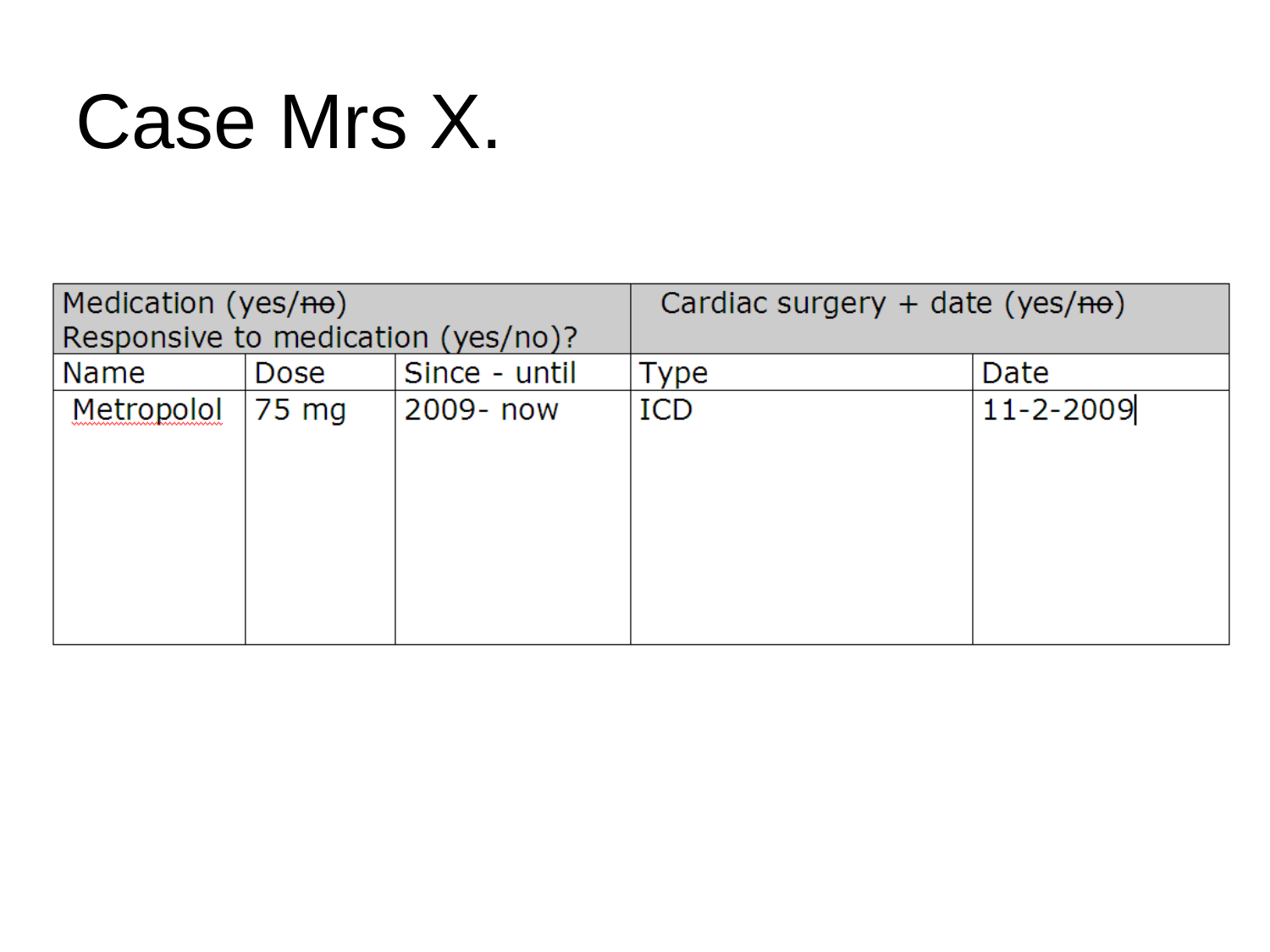

# Case Mrs X.

## Slide 23
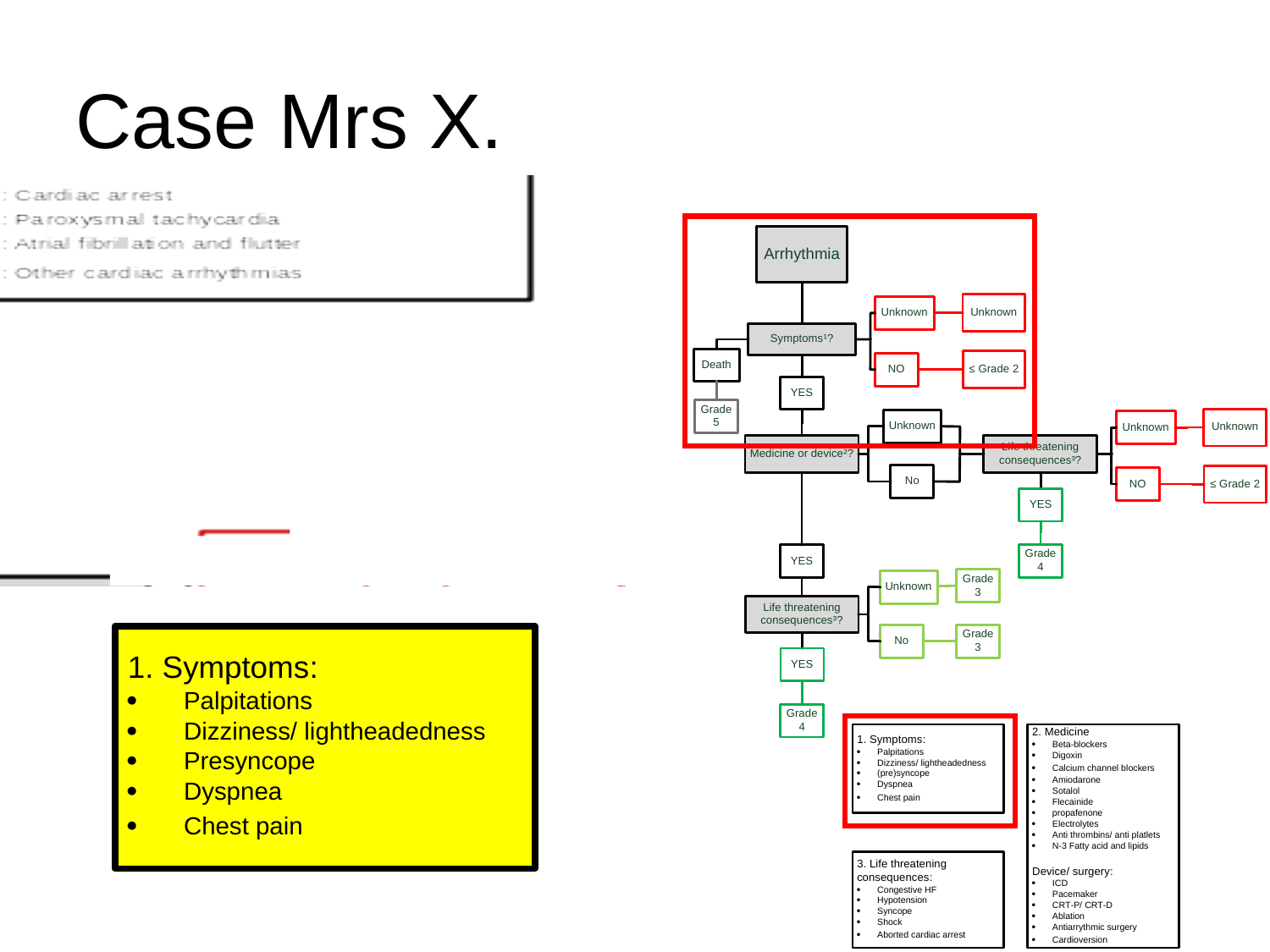

# Case Mrs X.

## Slide 24
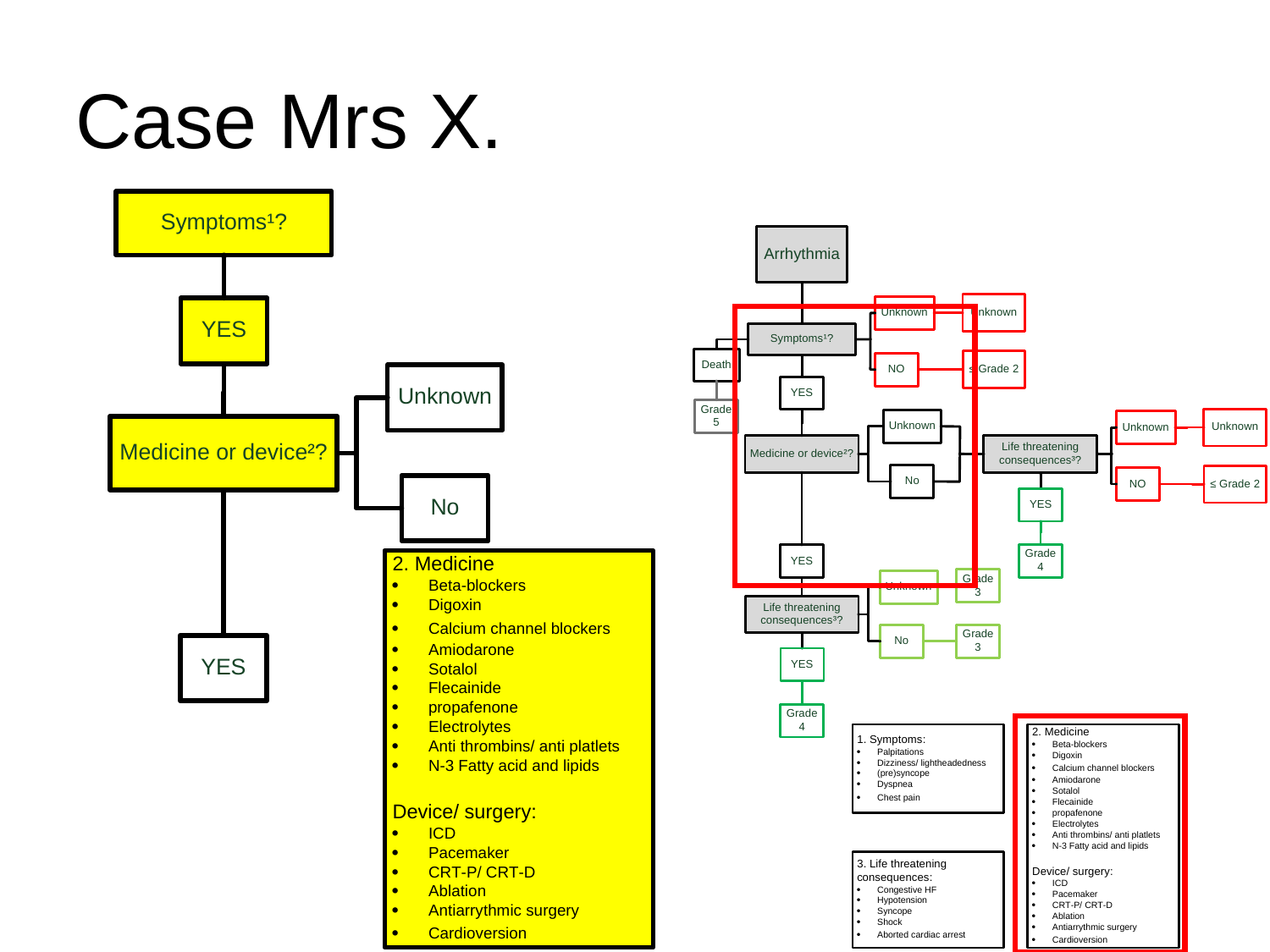

# Case Mrs X.

## Slide 25
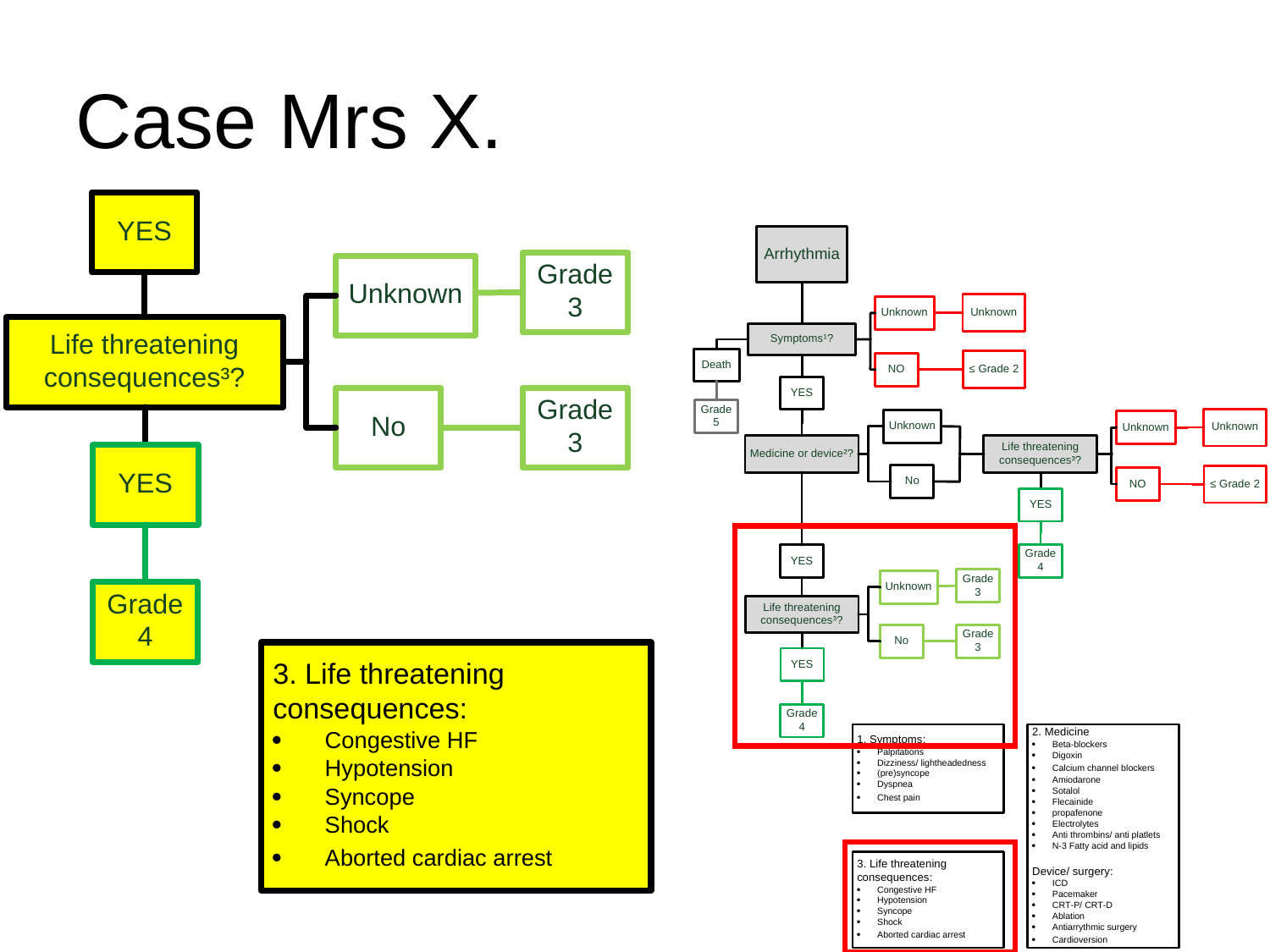

# Case Mrs X.

## Slide 26
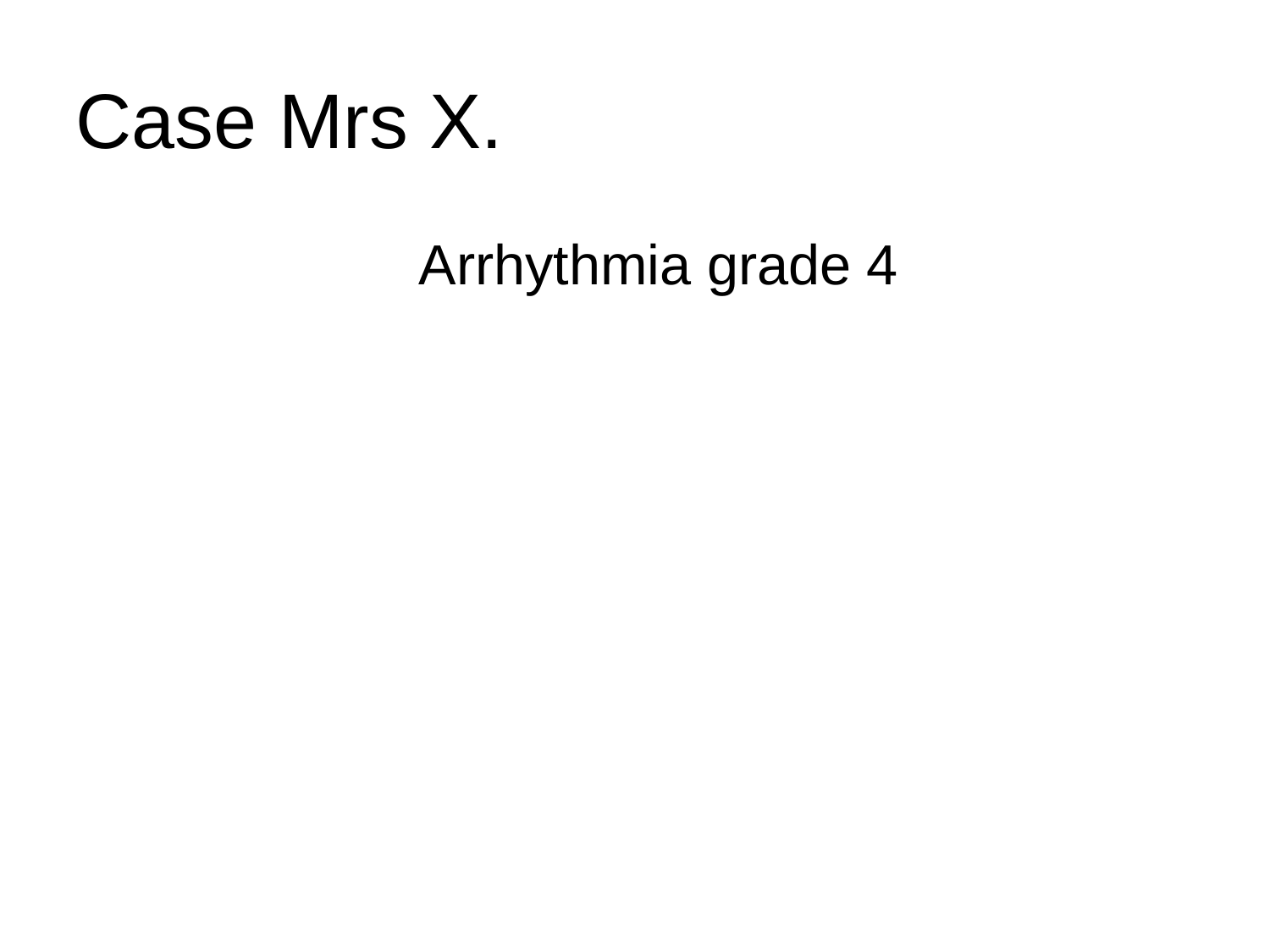

Case Mrs X.
# Arrhythmia grade 4
